# Artificial intelligence in cardiovascular pharmacotherapy: applications and perspectives

**DOI:** 10.1093/eurheartj/ehaf474

**Published:** 2025-07-15

**Authors:** Francesco Costa, Juan Jose Gomez Doblas, Arancha Díaz Expósito, Marianna Adamo, Fabrizio D’Ascenzo, Lukasz Kołtowski, Luca Saba, Guiomar Mendieta, Felice Gragnano, Paolo Calabrò, Lina Badimon, Borja Ibañez, Roxana Mehran, Dominick J Angiolillo, Thomas Lüscher, Davide Capodanno

**Affiliations:** Cardiology Department, University Hospital Virgen de la Victoria, Instituto de Investigación Biomédica de Málaga (IBIMA), Málaga 29010, Spain; Department of Biomedical and Dental Sciences and of Morphological and Functional Images, University of Messina, Messina 98122, Italy; Centro de Investigación Biomédica en Red en Enfermedades Cardiovasculares (CIBERCV), Instituto de Salud Carlos III, Madrid 28220, Spain; Cardiology Department, University Hospital Virgen de la Victoria, Instituto de Investigación Biomédica de Málaga (IBIMA), Málaga 29010, Spain; Centro de Investigación Biomédica en Red en Enfermedades Cardiovasculares (CIBERCV), Instituto de Salud Carlos III, Madrid 28220, Spain; Department of Medicine and Dermatology, University of Malaga, Málaga, Spain; Cardiology Department, University Hospital Virgen de la Victoria, Instituto de Investigación Biomédica de Málaga (IBIMA), Málaga 29010, Spain; Centro de Investigación Biomédica en Red en Enfermedades Cardiovasculares (CIBERCV), Instituto de Salud Carlos III, Madrid 28220, Spain; Institute of Cardiology, ASST Spedali Civili di Brescia, Department of Medical and Surgical Specialties, Radiological Sciences, and Public Health, University of Brescia, Brescia, Italy; Division of Cardiology, Cardiovascular and Thoracic Department, Città della Salute e della Scienza Hospital and University of Turin, Turin, Italy; 1st Department of Cardiology, Medical University of Warsaw, Warsaw, Poland; Department of Radiology, University of Cagliari, Cagliari, Italy; Department of Cardiology, Hospital de la Santa Creu i Sant Pau, Barcelona, Spain; Centro Nacional de Investigaciones Cardiovasculares (CNIC), Madrid, Spain; Department of Translational Medical Sciences, University of Campania ‘Luigi Vanvitelli’, Caserta 81100, Italy; Division of Clinical Cardiology, A.O.R.N. “Sant´Anna e San Sebastiano”, Caserta 81100, Italy; Department of Translational Medical Sciences, University of Campania ‘Luigi Vanvitelli’, Caserta 81100, Italy; Division of Clinical Cardiology, A.O.R.N. “Sant´Anna e San Sebastiano”, Caserta 81100, Italy; Centro de Investigación Biomédica en Red en Enfermedades Cardiovasculares (CIBERCV), Instituto de Salud Carlos III, Madrid 28220, Spain; Cardiovascular Program ICCC, Hospital de la Santa Creu i Sant Pau Research Institute, IIB-Sant Pau, CiberCV, Barcelona, Spain; Cardiovascular Research Chair, UAB, Barcelona, Spain; Centro de Investigación Biomédica en Red en Enfermedades Cardiovasculares (CIBERCV), Instituto de Salud Carlos III, Madrid 28220, Spain; Centro Nacional de Investigaciones Cardiovasculares (CNIC), Madrid, Spain; Cardiology Department, Instituto de Investigación Sanitaria-Fundación Jiménez Díaz (IIS-FJD,UAM), Madrid, Spain; Zena and Michael A. Wiener Cardiovascular Institute (R.M.), Icahn School of Medicine at Mount Sinai, New York, NY, USA; Division of Cardiology, University of Florida College of Medicine, Jacksonville, FL, USA; Royal Brompton & Harefield Hospitals and Cardiovascular Academic Group, King’s College, London, UK; Center for Molecular Cardiology, University of Zurich, Zurich, Switzerland; A.O.U. Policlinico ‘G. Rodolico—San Marco’, University of Catania, Catania 95123, Italy

**Keywords:** Artificial intelligence, Machine learning, Cardiovascular pharmacotherapy, Personalized therapy, Coronary artery disease, Thrombosis, Hypertension, Diabetes

## Abstract

Recent advances in artificial intelligence (AI) have shown great potential in improving cardiovascular pharmacotherapy by optimizing drug selection, predicting therapeutic efficacy and adverse effects, ultimately improving patient outcomes. Leveraging techniques like machine learning and *in silico* modelling, AI can identify populations likely to benefit from specific treatments, expedite novel drug discovery and reduce costs. Computational methods can also facilitate the detection of drug interactions and tailor interventions based on real-world data, supporting personalized care. Artificial intelligence–based approaches also show promise in streamlining clinical trial design and execution, leveraging on real-time data on patient responsiveness, enhancing recruitment efficiency. However, in order to fully realize these benefits, robust validation across diverse patient populations is necessary to ensure accuracy and generalizability. In addition, addressing concerns regarding data quality, privacy, and bias is equally critical to avoid exacerbating existing healthcare disparities. Scientific societies and regulatory agencies must ultimately establish standardized frameworks for data management, model certification, and transparency, to enable safe and effective integration of AI into clinical practice. This manuscript aims at systematically reviewing the current state-of-the-art applications of AI in cardiovascular pharmacotherapy, describing their current potential in guiding treatment decisions, refine trial methodologies and support drug discovery.

## Graphical Abstract

**Graphical Abstract:**
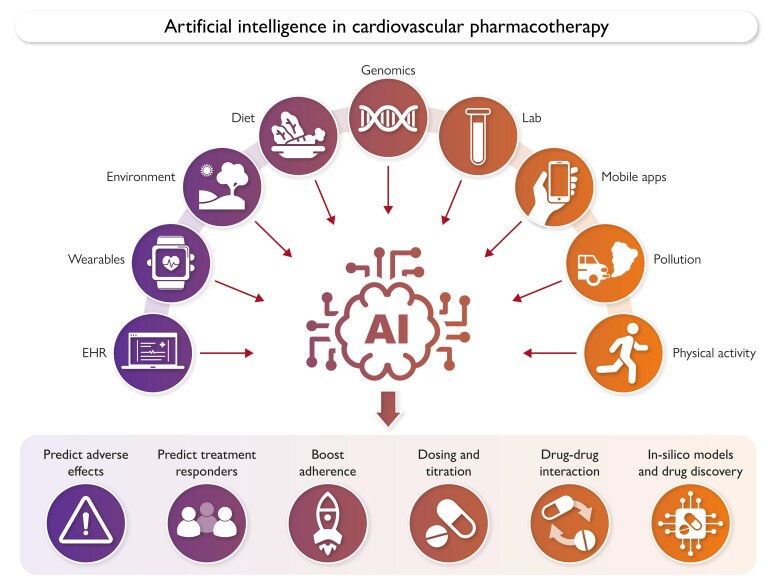
Source data and potential applications of artificial intelligence in cardiovascular pharmacotherapy. EHR, Electronic Health Records.

## Introduction

Artificial intelligence (AI) is an emerging science that simulates and enhances human intelligence to solve complex problems.^1^ Machine learning (ML), a foundational field within AI, enables computational systems to learn from data without explicit programming and has been applied in cardiovascular (CV) research for over 20 years. Unlike traditional statistical methods, which rely on explicit assumptions—such as linearity or normality—ML models can identify complex, non-linear patterns without rigid prior assumptions (see Supplementary data online, *Table S1*). ML effectively handles higher data complexity than typical statistical methods. For example, while traditional analyses might use single data-points per patient (e.g. diabetes status), ML manages extensive data containing millions of data-points, such as continuous physiological time-series (e.g. minute-level glucose monitoring), high-resolution imaging (e.g. pixels in a retinal fundus image), or the fusion of these multimodal data streams. This flexibility improves prediction but increases susceptibility to overfitting—learning dataset-specific noise rather than generalizable patterns. Hence, rigorous external validation is crucial for confirming generalizability and clinical utility of ML models. Advanced ML models such as neural networks (NN), and its more complex architectures, deep neural networks (DNN), have evolved significantly leveraging increased computational power and large, well-annotated, datasets. These now enable more sophisticated and complex learning, allowing for connections and inferences that were previously unattainable in cardiovascular medicine,^2–8^ investigating novel tools to diagnose low left ventricular ejection fraction (LVEF) using ECG readings,^9^ or detecting decompensated heart failure (HF) through patient voice recordings.^10^ Such developments have the potential to greatly expand our ability to diagnose and understand cardiovascular diseases in the future. Similarly, AI has the potential to optimize CV pharmacotherapy in multiple ways (*Graphical Abstract*). AI can identify patients who are more likely to respond to treatments, directing novel and costly therapies to those who will gain the most benefit. It can optimize drug dosing, predict adverse effects, and identify significant drug-drug interactions, particularly in the context of polypharmacy. Additionally, AI-platforms can enhance patient adherence by providing personalized reminders. Developments in *in silico* models and AI-driven drug discovery can accelerate the emergence of novel CV drugs by simulating biological processes and predicting compound efficacy, thus reducing the time and cost associated with traditional drug development methods.

In this manuscript, we provide a systematic review of the current state-of-the-art AI applications in cardiovascular pharmacotherapy by therapeutic domains (Search Strategy in Supplementary Methods), explore the potential of AI to support drug discovery, and discuss its associated challenges and opportunities.

## Evidence for AI in cardiovascular pharmacotherapy

### Hypertension

Hypertension remains one of the most undertreated cardiovascular (CV) risk factors. Despite the wide range of available therapeutic options, many patients continue to have inadequate blood pressure (BP) control, putting them at increased risk of adverse events.^11^ In this field, AI can be useful in supporting diagnosis as well as improving risk stratification and prognosis.^12,13^ Leveraging recent advances in wearable technologies for cuffless BP monitoring, AI-enabled devices hold significant promise for transforming remote hypertension management (see Supplementary Chapter  *S1*). However, due to current limitations in measurement accuracy and lack of standardization, traditional BP monitoring remains the recommended method for routine clinical assessment.^14,15^

In hypertension pharmacotherapy, AI-based models have the potential to personalize treatment by predicting the likelihood of achieving target BP. Herzog *et al*. developed a DNN using data from 16 917 newly diagnosed hypertensive patients to predict the most effective antihypertensive treatment combinations after 1 year. The model achieved a modest performance, outperforming physician practice.^16^ However, this model lacked external validation, and the population used to train the model mostly included white patients, limiting the external validity of this tool.

**Figure 1:**
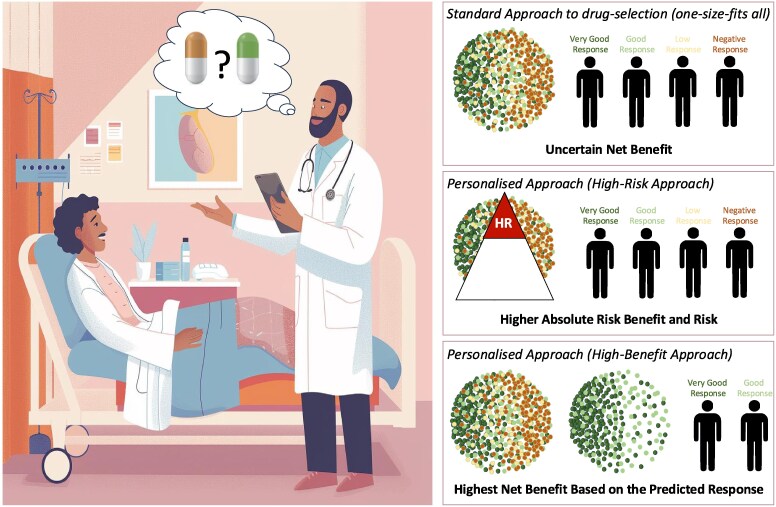
Potential for artificial intelligence to inform treatment decision-making for treatment personalization.

ML models are increasingly being developed to identify patients likely to derive the greatest benefit from intensive BP control, potentially refining treatment personalization. For instance, Oikonomou *et al*.,^17^ in a retrospective analysis using SPRINT trial data [non-diabetic patients randomized to intensive (<120 mmHg) vs. standard (<140 mmHg) BP targets], developed an ML model based on 8 clinical feature. Externally validated in the ACCORD BP study, this model successfully identified individuals predicted to benefit most from intensive control; this high-benefit group had a significantly lower risk of MACE compared to the predicted low-benefit group [adjusted hazard ratio (HR) 0.70 (95% CI 0.55–0.90) vs 1.05 (95% CI 0.84–1.32), *P* for interaction = .018]. Similarly, Inoue *et al*.^18^ directly compared guiding intensive antihypertensive treatment using traditional ‘high-risk’ stratification (based on Framingham/ACC/AHA scores) vs. a novel ML-driven ‘high-benefit’ approach predicting individual treatment effect (ITE). Analyzing 10 672 patients, they found the high-benefit strategy yielded a significantly greater average treatment effect (+9.36%) and was markedly more efficient. The Number Needed to Treat (NNT) to prevent one MACE was 11 with the high-benefit approach, compared to 45–61 using high-risk methods. Such ML-based ‘high-benefit’ approaches could mark a significant shift in treatment personalization (*Figure 1*). Traditionally, intensive therapies focused on patients with the highest baseline risk, assuming they gain the most benefit. However, this high-risk group may also be more vulnerable to treatment complications, potentially negating advantages. The proposed strategy leverages ML to predict individual responsiveness, aiming to target intensive treatments specifically towards those patients most likely to benefit from the treatment, rather than relying solely on baseline risk.

AI may also prove valuable in predicting drug-related side effects of hypertension treatments. For instance, Güven *et al.*^19^ tested ML models to predict renal adverse events associated with renin-angiotensin-aldosterone system inhibitors (RAASi) in a small cohort of 409 patients prior to initiation, achieving strong performance metrics. Similarly, Lin *et al*.^20^ developed an ML model to predict hypokalaemia in 25 326 hypertensive patients treated with diuretics; yet, both models lacked external validation. AI is also applied to drug repurposing. For example, Visanji *et al*.^21^ used IBM Watson for Drug Discovery™ to identify antihypertensives as potential Parkinson's disease (PD) treatments targeting alpha-synuclein oligomerization. A subsequent cohort study using the IBM MarketScan™ database linked certain antihypertensive combinations [angiotensin receptor II blockers + calcium channel blockers (CCB); angiotensin-converting enzyme inhibitors (ACEis) + diuretics] to significantly lower PD risk (HR = 0.55–0.60, *P* < .01), whereas alpha-blockers (alone or with CCBs) were associated with increased risk (HR = 1.81–3.17, *P* < .05).^21^ Importantly, such computationally generated hypotheses require experimental validation to confirm associations and avoid bias.

### Diabetes

AI’s potential in supporting diabetes diagnosis and care is well documented in recent literature.^22–25^ AI-driven closed-loop systems integrating continuous glucose monitors and smart insulin pumps have the potential to enhance treatment precision, advancing towards fully integrated artificial pancreas systems.^26–28^ Within the field of diabetes pharmacotherapy, AI may enhance personalized treatment strategies. For example, Villikudathil *et al*. developed an ML model using clinical, genomic, and proteomic data to predict good metformin response (i.e. achieving HbA1c ≤ 6.5%) in type 2 diabetes. Responders typically had lower creatinine and body weight. While the model predicted non-responders with 83% accuracy, it lacked external validation, which cannot exclude possible model overadjustment.^29^ Oikonomou *et al*. used data from the CANVAS trial to develop an ML model identifying diabetic patients deriving greater benefit from treatment with sodium glucose cotransporter 2 (SGLT2) inhibitors. They created a topological representation of the study population and trained an extreme gradient boosting (XGBoost) algorithm to predict the personalized effect of canagliflozin on CV events. This approach identified patient clusters more responsive to the treatment. The results indicated 37% of patients had a higher treatment response regarding MACE than the rest of the population. Validation in the CANVAS-Renal trial confirmed the model effectively identified patient phenotypes with significantly enhanced CV benefits from canagliflozin, showing an adjusted HR of 0.60 (95% CI 0.41–0.89) vs. 0.99 (95% CI 0.76–1.29) for the overall population (*P* interaction = .04).^30^ Edward *et al*. evaluated the impact of intensive vs. standard glycaemic control in 12 042 type 2 diabetes patients from the ACCORD and VADT trials by developing a causal forest ML model. The model, incorporating five variables (HbA1c index, eGFR, fasting glucose, age, and BMI), identified eight patient clusters with varying responses. Intensive glycaemic control effects ranged from a 5.1% absolute reduction to a 3.1% increase in MACE risk, depending on the patient cluster. These differences were independent of overall CV risk levels.^31^ With respect to the prediction of adverse events, multiple ML models have been proposed to predict hypoglycaemia risk during hospitalization,^32^ lower extremity amputations,^33^ or MI,^34^ in patients treated with different oral antidiabetic drugs.

Finally, *in silico* models are valuable tools in developing new drugs and improving existing ones. For instance, they have been used to study the binding mechanisms of SGLT2 inhibitors,^35^ and to identify new fingerprints—small molecular fragments that can serve as building blocks for drug design for this drug class.^36^  *In silico* analyses have also been used to enhance dipeptidyl peptidase-4 (DPP-4) inhibitors, aiming to reduce side effects from off-target DPP-8/9 inhibition. Hermansyah *et al*.^37^ performed quantitative structure-activity relationship (QSAR)-based virtual screening of over 10 million molecules, identifying 2716 potential candidates with reduced off-target inhibition risk. Subsequent docking analysis identified a novel, highly selective DPP-4 inhibitor candidate with minimal DPP-8/9 activity, offering a promising route to safer DPP-4 inhibitors.

### Dyslipidaemia

AI application in the pharmacotherapy of dyslipidaemias mostly focused on models supporting statin use. Already in 2002, a small study by Moon *et al*.^38^ implemented a NN to predict appropriate dosing of statins in a lipid clinic. Years later, AI was applied to improve treatment plans in patients at high cardiovascular risk who were already receiving statin therapy,^39^ or suggesting an optimized initial treatment prescriptions to limit adverse events and treatment non-adherence.^40^ Chi *et al*. developed a ML model to predict the optimal statin treatment plan, including the choice of drug type and dosage, aimed at minimizing adverse events and minimizing non-adherence in 38 214 patients with a recent cardiovascular event. They trained a NN using 70 baseline clinical characteristics, which led to significantly lower risks of statin-associated symptoms (10.87% vs. 14.82%) and treatment discontinuation compared to standard practice (55.27% vs. 60.95%, *P* < .00001).^40^ Sarraju *et al*.^41^ sought to understand the drivers of statin underprescription in a large cohort of 56 530 patients from the Stanford Health Care Alliance. They developed a DL-based natural language processing (NLP) approach to systematically track statin nonuse and reasons thereof within unstructured electronic health record (EHR) data. Among 21 508 patients without statin prescriptions, only 3929 (18%) had any documentation discussing statin use or nonuse, respectively in their EHRs. The model effectively identified statin nonuse with an AUC of 0.94 (95% CI: 0.93–0.96) and reasons for nonuse with an AUC of 0.88 (95% CI: 0.86–0.91). The key reasons identified included patient-level factors, such as side effects and patient preferences, and clinician-level factors, such as guideline-discordant practices. Notably, these reasons varied by the type of atherosclerotic cardiovascular disease and patient race or ethnicity. Finally, Horne *et al*.^42^ prospectively assessed in a randomized controlled trial an AI-driven intervention using personalized electronic ‘nudges’—messages that, unlike simple reminders, subtly guide choices by altering the decision environment, making the preferred options more appealing—to improve statin adherence. Compared to controls at 12 months, the nudge group achieved significantly higher adherence [proportion of days covered (PDC): 0.742 vs. 0.639, *P* = .042; % adherent (PDC ≥ 80%): 66.3% vs. 50.5%, *P* = .036] and showed non-significantly lower MACE (6.7% vs. 10.8%, *P* = .44).

As novel treatments target Lipoprotein(a) [Lp(a)], AI tools offer significant potential for guiding pharmacotherapy where solutions are currently lacking. Scalable AI screening models using EHR data can identify patients likely to have high Lp(a), thus prioritizing currently scarce large-scale testing. For example, the ML-based ARISE score uses common clinical features to optimize Lp(a) screening, reducing the number needed to test by up to 67.3%.^43^ Deployment in a large EHR, including 100 000 randomly selected patients, confirmed this potential prospectively: the score was computable in roughly one-third of patients, identifying a high-probability cohort (7.5% of total) with 1.41- to 1.87-fold higher prevalence of Lp(a), thereby informing targeted screening strategies.^44^

### Thrombosis

Anticoagulant therapy presents ongoing challenges in medical practice, prompting the development of numerous clinical prediction models aimed at improving treatment adherence, prevent adverse events, and optimize maintenance dose.^45^ Choi *et al*.^46^ trained several ML models to predict warfarin discharge doses using clinical data extracted from EHR within the first 2 days of hospitalization. The performance of the models was evaluated by comparing them against physician predictions. The results showed that all four trained models—XGBoost, artificial NN, random forest, and linear regression—outperformed physician judgment in internal validation, demonstrating their superior accuracy in predicting optimal warfarin discharge doses, but not in the external validation set. Similarly, Lee *et al*.^47^ developed a prediction model and a decision support system for determining warfarin maintenance doses. Their algorithm utilized dense and recurrent NN trained on data from 19 719 patients to forecast the international normalized ratio (INR) level on the fifth day after initiating warfarin therapy. The AI model outperformed physician judgment, accurately predicting INR within ±0.3 of the actual value in 84.0% of cases (10 650/12 673), compared to 81.9% (1647/2000) for expert physicians (*P* = .014); yet, no external validation was performed.^47^ These findings highlight the significant potential of AI-based tools for optimizing warfarin dosing in daily clinical practice. By utilizing readily available clinical parameters, ML models can assist clinicians by forecasting INR trajectories and predict both initial dose selection and subsequent adjustments, generating patient-specific recommendations suitable for direct integration into EHRs. However, rigorous external validation is essential. A notable gap currently exists between the internal performance of these models and their effectiveness in external validation studies, limiting their immediate applicability and scalability to populations beyond the original development cohort. With respect to risk prediction, Goto *et al*.^48^ developed a model to predict clinical outcomes up to 1 year in patients with atrial fibrillation (AF) receiving vitamin K antagonists, utilizing data from the GARFIELD-AF registry. The NN was trained on INR measurements collected within the first 30 days of treatment and clinical outcomes recorded from days 31 to 365. The model achieved c-statistics of 0.75 for major bleeding, 0.70 for stroke or systemic embolism (SE), and 0.61 for all-cause mortality. Notably, the AI model outperformed the traditional time in therapeutic range evaluation, offering a more accurate approach to risk prediction in AF patients on anticoagulation therapy. Several ML models have been developed to assess haemorrhagic risk in patients taking direct oral anticoagulants (DOAC).^49,50^ Huang *et al*. analyzed data from the RELY trial, applying integrated ML methods, to identify key variables for predicting vascular events in patients treated with dabigatran 110 mg, and bleeding in those treated with dabigatran 150 mg. Random forest model achieved AUCs of 0.76 for the 110 mg dose and 0.75 for the 150 mg dose, while XGBoost had AUCs of 0.71 for the 110 mg dose and 0.76 for the 150 mg dose, outperforming logistic regression.^50^ The two best models identified ten critical variables and generated tree-shaped rules for predicting vascular and bleeding events, highlighting the potential of risk models of personalizing DOAC dose.^50,51^

The use of AI to identify drug–drug interactions in patients treated with warfarin has also been explored in a data-mining study.^52^ A random forest model was employed to predict changes in INR levels following new prescriptions in previously INR-stable warfarin-treated patients with nonvalvular AF. The analysis identified two drug groups with known interactions—β-lactamase-resistant penicillins and carboxamide derivatives—as well as three antithrombotic agents that were associated with a decrease in INR. Additionally, the model rediscovered six drug groups with known interactions that caused an increase in INR, including class III antiarrhythmics, opioids (e.g. tramadol), glucocorticoids, and triazole derivatives. Notably, antipropulsives were identified as having a previously unknown association with increased INR.

### Coronary artery disease

Managing patients with coronary artery disease (CAD) remains challenging, particularly in stratifying risks for ischaemic and bleeding events and mortality to guide treatment decisions.^53–59^

Recently, new AI-risk-tools have been developed to provide individualized and accurate outcome predictions.^60,61^ In 2020, Gibson *et al*.^60^ reported the first study to compare ML with traditional models for predicting ischaemic and bleeding events after ACS. The ML model, trained on 24 178 ACS patients and including 48 variables, showed superior discrimination compared to the TIMI risk score (c-index: 0.73 vs. 0.49; *P* = 0.001) but not to a stepwise logistic regression model, thus not demonstrating significant improvement over a traditional approach.^60^ More recently, D’Ascenzo *et al*.^61^ developed the ML-based PRAISE score to predict 1-year all-cause death, recurrent MI, and major bleeding post-ACS. Trained on 19 826 ACS patients, it was externally validated in a pooled population of 3444 patients from the SECURITY trial and real-world registries, demonstrating good predictive performance at 1-year (AUC: death 0.92; 95% CI: 0.90–0.93—MI 0.81; 95% CI: 0.76–0.85—major bleeding 0.86; 95% CI: 0.82–0.89).^61^ Subsequently, Patti *et al*.^62^ evaluated DAPT regimens in 21 960 ACS patients stratified by PRAISE score risk. Compared to potent P2Y12i, clopidogrel was associated with an increased risk of recurrent MI among patients with low-to-moderate bleeding risk and low-to-moderate ischaemic risk, whereas was associated to a higher risk of death and recurrent MI among those at low-to-moderate bleeding risk and high ischaemic risk. In contrast, in patients at high bleeding risk, clopidogrel was not associated with an excess of ischaemic events compared to potent P2Y_12_ inhibitors, independent of ischaemic risk.^62^ While the PRAISE score shows potential in guiding DAPT intensity after ACS, a comparison with existing risk scores remains needed, as does prospective validation in randomized trials.^61^ Risk prediction tools developed using ML techniques to guide pharmacotherapy in CAD patients are inherently different from traditional models. ML models captures complex, non-linear interactions and latent patterns in high-dimensional data, resulting in high discriminative performance. However, could be less transparent, difficult to interpret, and more prone to overfitting. In contrast, traditional models based regression and a limited set of predefined predictors, offer greater interpretability and ease of implementation, but with reduced flexibility in modeling more complex relationships. These differences highlight a trade-off between predictive accuracy and clinical usability.

### Heart failure

HF represents a major challenge in CV medicine^63^ and is expected to increase due to new technologies enabling early diagnosis and increased life expectancy.^63,64^

The application of AI in this context holds great potential for early diagnosis, characterization of HF phenotypes, stratification HF severity and optimization of treatment. Recent studies reported an excellent performance of AI-tools based on ECG, echocardiography, heart sounds and EHR derived data, to support HF diagnosis.^65–68^ Similarly, several ML algorithms have also been proposed for the prognostic stratification of HF patients.^69–71^ These tools may be of great utility in specific subgroups of HF patients in which phenotypic characterization and treatment remains challenging.

#### Heart failure with preserved ejection fraction

HF with preserved ejection fraction (HFpEF) is a heterogeneous condition with limited therapeutic options.^72^ AI may offer a hypothesis-generating framework for clinical trials guiding tailored treatments by identifying distinct HFpEF endotypes based on clinical profiles, outcomes, and potential therapy responses.^73–76^ Shah *et al.*^74^ identified three phenotypes of HFpEF with the highest-risk group having chronic kidney disease, AF, and NTproBNP levels. Przewlocka-Kosmala *et al.*^77^ used automated hierarchical clustering based on echocardiographic variables to identify a subgroup of patients with impaired diastolic and/or chronotropic reserve that had worse prognosis. AI has also been applied to predict drug response. Kresoja *et al*.^78^ used cluster analysis on Aldo-DHF trial data, identifying a subgroup (38% of patients) that responded to spironolactone via improved E/e’ ratio. An XGBoost classifier based on these findings, when validated in the TOPCAT cohort, confirmed spironolactone significantly reduced the primary outcome only among predicted ‘responders’ (35% of cohort), not in ‘non-responders’ (65%).

#### Heart failure with reduced ejection fraction

While effective HFrEF therapies exist, predicting individual pharmacological response remains challenging. Mohebi *et al*. created an ML model predicting LVEF change after sacubitril/valsartan therapy; it showed good internal validation for identifying non-improvers but lacked external validation. While such approach could potentially inform early decisions for cardiac resynchronization therapy or implantable cardioverter-defibrillators, prospective validation with contemporary guideline-directed medical therapy remain essential.^79^ Karwath *et al*. applied NN and hierarchical clustering to 15 659 HFrEF patients from 9 randomized, placebo-controlled trials evaluating β-blocker response. They found that while β-blockers were largely effective among patients in sinus rhythm with a reduction in mortality ranging from 26% to 46%, a specific cluster of older patients with fewer symptoms showed no significant benefit from β-blockers (OR 0.86, 95% CI 0.67–1.10; *P* = .22). Conversely, while β-blockers proved largely ineffective among patients with AF, one specific cluster of younger patients with AF obtained a significant prognostic benefit, with a 43% reduction in mortality (OR 0.57, 0.35–0.93; *P* = .023).^80^ Furthermore, in a population of 1802 patients with HFrEF from the BIOSTAT-CHF study, Tromp *et al*. identified six HFrEF endotypes solely based on biomarker profiles using unsupervised cluster analysis. These patient clusters experienced significantly different outcomes and response to guideline directed medical therapy, with specific groups benefitting from uptitration of β-blockers and RAASi, and others showing potential harm.^81^ Defining patient endotypes, based on biomarkers or clinical features, appears promising for predicting therapy response but warrants prospective evaluation.

#### Acute heart failure

Multiple AI-based models were developed to evaluate the probability of patient deterioration,^82^ readmission,^83^ early and late mortality in acute heart failure.^83–85^ However, less evidence exists for models guiding pharmacological treatment. In this context, Mercier *et al*.^86^ generated models predicting diuretic resistance in HF patients using 21 variables (including creatinine, SBP, chloride, age, sex). Additional technologies, such as implantable pulmonary pressure monitors, hold promise when combined with AI for advanced data analysis and treatment adjustments. The LINK-HF trial demonstrated that ML analysis of remote monitoring data could predict HF readmission with high accuracy (87.5% sensitivity, 85% specificity).^87^

### Arrhythmia

Although most evidence for the application of AI in arrhythmias focuses on the automated ECG diagnosis of AF,^88,89^ and rhythm management,^90^ an increasing number of studies have been published on the evaluation of pharmacotherapy with antiarrhythmic drugs.

#### AF

AI models in AF have been developed to forecast the effects of antiarrhythmic drugs based on electrophysiological patterns.^91^ For amiodarone, AI applications have been utilized to closely monitor patients during treatment, helping to mitigate adverse effects of the drug.^92^ Lu *et al.*^93^ developed an XGBoost model to predict amiodarone-induced thyroid dysfunction, achieving a detection rate of 71.3%. Within the multicenter AADGEN study, multiple ML algorithms were trained to monitor dofetilide initiation, predicting dosing decisions with 96% accuracy.^94^ Additionally, researchers from the Mayo Clinic developed a DL model that used ECG data to predict dofetilide plasma concentrations.^95^

#### Long QT Syndrome

Numerous studies have evaluated AI's usefulness in drug-induced long QT syndrome. Prifti *et al*.^96^ aimed to improve prediction of torsade de pointes (TdP) in patients with congenital (cLQTS) or drug-induced (diLQTS) long-QT syndromes using convolutional neural network (CNN) models analyzing ECG alterations induced by sotalol, a known IKr blocker. CNN models trained on 10-second ECG data from healthy individuals exposed to sotalol recognized the distinct ECG effects of drug exposure (as a proxy for IKr blockade). The models were then tested in separate cohorts of patients with cLQTS or TdP episodes. Results showed CNN-based ECG interpretation significantly outperformed traditional QTc measurements for identifying sotalol exposure (ROC-AUC = 0.98 vs 0.72, *P* < .001), with comparable performance on both 8-lead and single-lead ECGs. The CNN models also accurately distinguished cLQTS from healthy controls, particularly cLQT2 (ROC-AUC = 0.9), which shares sotalol's mechanism. Moreover, these models improved TdP event prediction, even after adjusting for QTc values and known TdP risk drugs. Analyses of the CNN models revealed specific ECG features, notably the J-T peak interval, as critical markers for identifying sotalol-induced ECG changes, highlighting model interpretability and clinical relevance.^96^

## Artificial intelligence for *in silico* models and drug discovery

**Figure 2:**
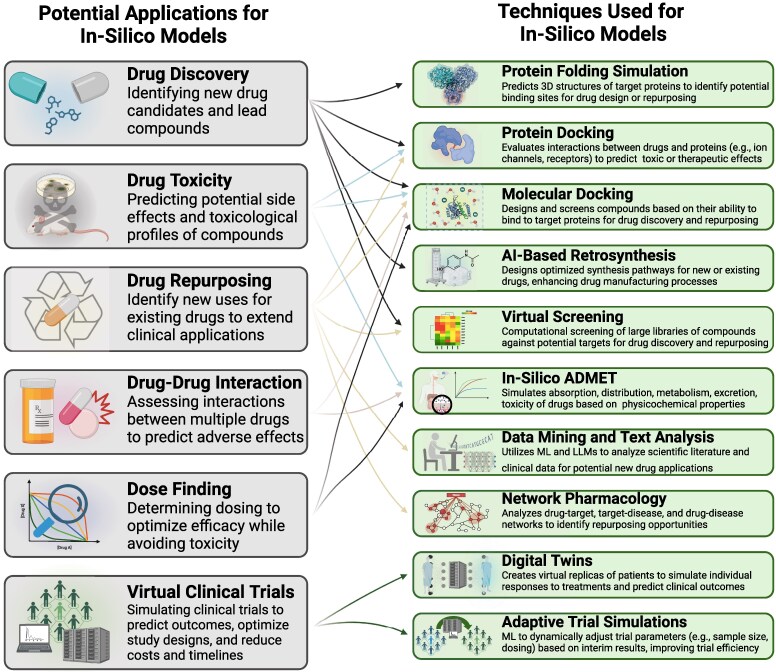
Potential applications and techniques used for *in silico* models in cardiovascular pharmacotherapy. LLM, Large Language Model; ML, machine learning.

The process of drug discovery is highly expensive and time-consuming; current estimates suggest bringing a novel molecule to market requires approximately one billion dollars in funding and over 10 years of work.^97,98^ AI has great potential to streamline this process, offering efficiencies that reduce associated timelines and costs, both at a preclinical stage empowering *in silico* models, and at a clinical stage improving clinical trial design and execution (*Figure 2*). Classically, *in silico* models have been used for drug repurposing,^99^ protein–protein docking, *de novo* protein design,^100^ or adverse events prediction, among others. Novel AI-based iterations allow for faster and more precise drug discovery and development. Random forests have been utilized to identify hit and lead compounds, exemplified in QSAR models.^101^ Additionally, synthesizing lead compounds is enhanced through NN retrosynthesis algorithms. These algorithms, combined with best-chance trees and the input of vast amounts of accumulated data and rules, can generate synthesis pathways with >90% accuracy.^102^ More recently, the use of DNN play a crucial role in optimizing drug structure and properties,^103^ assisting in the 3D structure prediction of targeted proteins and understanding protein–ligand interactions.^104^ For example, the new version of AlphaFold, an AI model developed by Google’s DeepMind that predict the folding properties of known proteins, demonstrated significantly greater accuracy in modelling protein-ligand interactions, protein-nucleic acid, and antibody-antigen interactions compared to current state-of-the-art tools.^105,106^ More recently, the same company released AlphaProteo, a DNN based model able to design totally novel proteins from scratch based on desired binding targets.^107^ These novel and revolutionary tools based on a DL frameworks have potential to revolutionize future drug discovery.

Other relevant applications of AI and DNN are in the field of drug repurposing. These technologies can identify new uses for existing drugs, potentially allowing them to skip early-phase trials.^108^ Finally, AI has the potential to significantly enhance the design and execution of randomized trials. Advanced tools have been developed to predict the success of a trial based on the drug molecule, target disease, and patient eligibility criteria.^98^ Generative AI models can summarize data from numerous similar trials and assist in trial design by suggesting optimized study inclusion criteria and outcomes. AI applications can also expedite patient recruitment. For instance, the application TrialPathfinder evaluates completed clinical trials and assesses how adjusting participation criteria affects HR.^109^ In a previous study, adjusting inclusion criteria as suggested by this application would have doubled the number of eligible patients without modifying the HR. Adaptive models can progressively adjust patient inclusion characteristics, favouring the inclusion of those patients with the highest probability of responding to treatment, thereby reducing the sample size needed. Oikonomou *et al.* proposed a ML-based method that uses phenomapping strategies to highlight clusters of patients with specific characteristics who respond well to the treatment under investigation. Applied retrospectively to two prior RCTs, this strategy was able to reduce sample size by 15–17% while preserving the original average treatment effect, thereby maximizing RCT enrolment efficiency.^110^ The implementation of digital twins offers another innovative approach. Based on an experimental patient’s data at the start of a trial, researchers can use a digital twin to predict how the same patient would have progressed in the control group and compare outcomes.^111^ This method typically reduces the number of control patients needed by between 20% and 50%, benefiting patients by lowering the likelihood of receiving a placebo.^98^

## Present and future of artificial intelligence in cardiovascular pharmacotherapy

**Figure 3.**
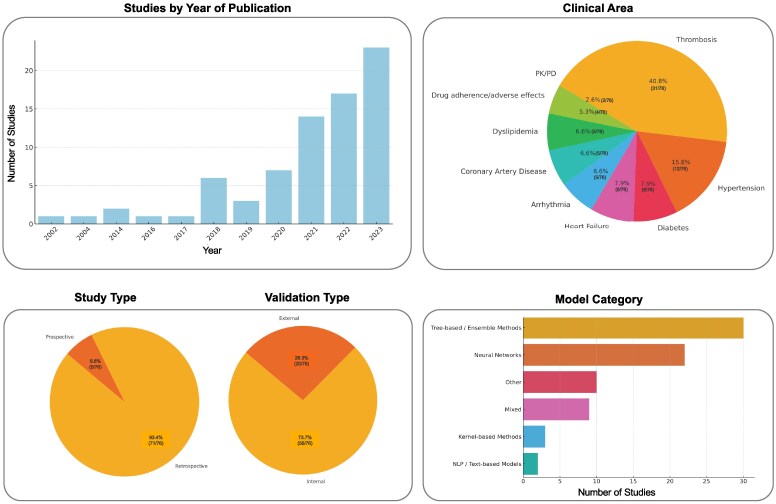
Summary of study characteristics for artificial intelligence tools in cardiovascular pharmacotherapy. Studies are reported according to the year of publication, clinical area, study, validation and machine learning model type implemented. Details regarding search methods and list of studies included are available in the Supplementary data online, appendix. For study year of publication and model type the absolute number of studies included is reported. For study clinical area, study and validation type, the relative percentage is reported. NLP, natural language processing; PK/PD, pharmacokinetic/pharmacodynamic.

An overall summary of the body of evidence examined in this review is presented in *Figure 3* and Supplementary data online, *Table S2*. During the past 5 years, studies of AI in cardiovascular pharmacotherapy have risen sharply, with an even distribution across therapeutic domains and a relative concentration in thrombosis and hypertension. Yet, only a small fraction of these models has been assessed prospectively or externally validated in independent cohorts. Because ML algorithms are inherently prone to over-fitting, rigorous external and prospective validation—ideally embedded in randomized clinical trials—is essential to demonstrate clinical utility and to support adoption in routine practice. Consequently, few models are currently ready for real-world implementation.

### Pitfalls and limitations of artificial intelligence

While AI holds significant potential for CV care, key considerations must be addressed. First, AI model effectiveness hinges on high-quality, unbiased data. To prevent AI from perpetuating or amplifying existing disparities and unintentionally harming underrepresented or vulnerable patient groups, addressing diversity, equity, and inclusion (DE&I) in its development and deployment is crucial.^112,113^ This necessitates training and validating AI models on data that accurately reflect the full spectrum of patient populations, encompassing variations in age, sex, ethnicity, socioeconomic background, and comorbidities. Such comprehensive data ensures adequate performance and reliability across all patient groups, not just the majority. Equally important is the equitable deployment of these tools to support fair access to care in cardiovascular medicine, thereby helping to reduce, rather than widen, existing gaps in care. Second, integrating AI technologies, including large language models, raises significant privacy concerns. To comply with strict privacy regulations (e.g. GDPR, HIPAA), local integration within the hospital's secure IT environment is necessary. This prevents transferring sensitive patient data to external servers, mitigating privacy risks. Crucially, local integration allows technologies like natural language processing to securely process rich, often underutilized, unstructured EHR data (e.g. clinical notes) alongside structured data.^114^ Finally, as many AI-based models currently lack rigorous validation, establishing standardized frameworks for data quality, validation and registration is essential, and should be a priority for scientific societies, guideline committees, and regulatory agencies. Such frameworks would set model quality standards and determine the evidence level required for clinical adoption, analogous to processes for other medical devices.

### Future outlook

Notwithstanding current limitations and challenges, rapid methodological advances make it likely that a growing number of robust, clinically useful tools will soon emerge. As *in silico* platforms enlarge the pipeline of compounds reaching human studies, shorten the interval from dose-finding to late-phase trials, and accelerate drug-repurposing initiatives, these are poised to markedly broaden our pharmacological armamentarium. Improving medication adherence is another, mature use-case for AI, already well explored in prospective studies. Novel smart sensors, wearables, and extended-reality interfaces equipped with AI can reduce non-adherence, a major modifiable contributor to adverse outcomes (see Supplementary Chapter  *S2*).^115^ Finally, AI will underpin the next phase of treatment personalization. Integrating genomic data, clinical characteristics, continuous physiological monitoring (e.g. glucose, lactate, electrocardiogram and blood pressure), circulating biomarkers, imaging, and environmental exposures will generate complex multimodal data streams. Algorithms capable of synthesizing these inputs to support dynamic therapy adjustment can be deployed across multiple points of care—from EHR systems that assist clinicians to patient-facing mobile applications that enable self-management.

## Conclusions

In conclusion, integration of AI into cardiovascular pharmacotherapy is revolutionizing disease management by advancing drug development, personalizing treatment, optimizing dosing, and predicting adverse reactions. However, gaps remain in specific areas, and rigorous external validation of these models is crucial to confirm their generalizability and support broader adoption. Continued research is essential to fully harness AI's potential, leading to improved patient outcomes and more efficient healthcare. Careful attention must be paid to data quality, privacy concerns, and the need for robust validation frameworks to ensure that AI tools are safely and effectively integrated into clinical practice—a priority that guideline committees and regulatory agencies should address to guide clinical implementation.

## Supplementary Material

ehaf474_Supplementary_Data

## Declarations

### Data Availability

The data underlying this article are available in the article and in its online supplementary material.
