# Supplementary material for: Artificial intelligence in cardiovascular pharmacotherapy: applications and perspectives
Source: Eur Heart J. 2025 Jul 15;46(37):3616–27. doi: 10.1093/eurheartj/ehaf474 (PMC12488325; doi:10.1093/eurheartj/ehaf474)
Supplement: ehaf474_Supplementary_Data [file ehaf474_supplementary_data.docx]

**SUPPLEMENTARY APPENDIX**

Artificial intelligence in cardiovascular pharmacotherapy: current applications and future perspectives

***Costa et al.***

**Supplementary Methods: Search strategy**

*Aim*

To evaluate original research articles implementing artificial intelligence methods or algorhythms to inform or modify cardiovascular pharmacotherapy strategy.

*Search strategy*

Databases searched: Medline form 2000 to September 2024, using the following search strategies:

SEARCH 1: “CARDIOVASCULAR PHARMACOTHERAPY AND AI” (N=95)

(("Cardiovascular Diseases"[MeSH Terms]) AND ("Artificial Intelligence"[MeSH Terms])) AND (("Antihypertensive Agents"[MeSH Terms] OR "Anti-Arrhythmia Agents"[MeSH Terms] OR "Anticoagulants"[MeSH Terms] OR "Platelet Aggregation Inhibitors"[MeSH Terms] OR "Hydroxymethylglutaryl-CoA Reductase Inhibitors"[MeSH Terms] OR "Vasodilator Agents"[MeSH Terms] OR "Diuretics"[MeSH Terms] OR "Adrenergic Beta-Antagonists"[MeSH Terms] OR "Calcium Channel Blockers"[MeSH Terms] OR "Angiotensin-Converting Enzyme Inhibitors"[MeSH Terms] OR "Angiotensin Receptor Antagonists"[MeSH Terms] OR "Cardiotonic Agents"[MeSH Terms] OR "Digitalis Glycosides"[MeSH Terms] OR "Sodium-Glucose Transporter 2 Inhibitors"[MeSH Terms] OR "Glucagon-Like Peptide-1 Receptor Agonists"[MeSH Terms] OR " Proprotein Convertase 9 inhibitors"[MeSH Terms] OR " Endothelin Receptor Antagonists"[MeSH Terms] OR " Phosphodiesterase 5 antagonists"[MeSH Terms] OR " Guanylate Cyclase Stimulators"[MeSH Terms]) AND "Cardiovascular Diseases/drug therapy"[MeSH Terms])

SEARCH 2: “CARDIOVASCULAR DRUGS AND AI”: (N=740)

(machine learning OR artificial intelligence) AND (Lisinopril OR Enalapril OR Ramipril OR Captopril OR Quinapril OR Perindopril OR Fosinopril OR Benazepril OR Trandolapril OR Moexipril OR Losartan OR Valsartan OR Telmisartan OR Irbesartan OR Candesartan OR Olmesartan OR Eprosartan OR Azilsartan OR Metoprolol OR Atenolol OR Propranolol OR Bisoprolol OR Carvedilol OR Nebivolol OR Sotalol OR Timolol OR Labetalol OR Nadolol OR Amlodipine OR Diltiazem OR Nifedipine OR Verapamil OR Felodipine OR Nicardipine OR Isradipine OR Lacidipine OR Nimodipine OR Bepridil OR Hydrochlorothiazide OR Furosemide OR Spironolactone OR Chlorthalidone OR Triamterene OR Amiloride OR Bumetanide OR Torsemide OR Indapamide OR Metolazone OR Warfarin OR Heparin OR Enoxaparin OR Dalteparin OR Fondaparinux OR Bivalirudin OR Dabigatran OR Rivaroxaban OR Apixaban OR Edoxaban OR Betrixaban OR Aspirin OR Clopidogrel OR Prasugrel OR Ticagrelor OR Dipyridamole OR Ticlopidine OR Cangrelor OR Vorapaxar OR Eptifibatide OR Tirofiban OR Atorvastatin OR Simvastatin OR Rosuvastatin OR Pravastatin OR Lovastatin OR Fluvastatin OR Pitavastatin OR Cerivastatin OR Amiodarone OR Sotalol OR Dronedarone OR Flecainide OR Propafenone OR Lidocaine OR Mexiletine OR Quinidine OR Disopyramide OR Procainamide OR Sacubitril/Valsartan OR Spironolactone OR Eplerenone OR Nitroglycerin OR Isosorbide Mononitrate OR Isosorbide Dinitrate OR Erythrityl Tetranitrate OR Pentaerythritol Tetranitrate OR Canagliflozin OR Dapagliflozin OR Empagliflozin OR Sotagliflozin OR semaglutide OR liraglutide OR Dulaglutide OR Exenatide OR tirzepatide OR Bosentan OR Ambrisentan OR Sildenafil OR Tadalafil OR Epoprostenol OR Treprostinil OR Iloprost OR Riociguat OR Selexipag OR Nifedipine OR Diltiazem OR Amlodipine OR Alirocumab OR Evolocumab OR Inclisiran)

SEARCH 3: “RISK SCORES AND MODELS FOR CARDIOVASCULAR DRUGS”: (N=198)

((risk score OR decision-making OR decision tool OR risk algorithm OR prediction tool OR risk model) OR ("Decision Support Systems, Clinical"[MeSH Terms] OR "Risk Assessment"[MeSH Terms] OR "Models, Theoretical"[MeSH Terms] OR "Predictive Value of Tests"[MeSH Terms])) AND ("Artificial Intelligence"[MeSH Terms]) AND (Lisinopril OR Enalapril OR Ramipril OR Captopril OR Quinapril OR Perindopril OR Fosinopril OR Benazepril OR Trandolapril OR Moexipril OR Losartan OR Valsartan OR Telmisartan OR Irbesartan OR Candesartan OR Olmesartan OR Eprosartan OR Azilsartan OR Metoprolol OR Atenolol OR Propranolol OR Bisoprolol OR Carvedilol OR Nebivolol OR Sotalol OR Timolol OR Labetalol OR Nadolol OR Amlodipine OR Diltiazem OR Nifedipine OR Verapamil OR Felodipine OR Nicardipine OR Isradipine OR Lacidipine OR Nimodipine OR Bepridil OR Hydrochlorothiazide OR Furosemide OR Spironolactone OR Chlorthalidone OR Triamterene OR Amiloride OR Bumetanide OR Torsemide OR Indapamide OR Metolazone OR Warfarin OR Heparin OR Enoxaparin OR Dalteparin OR Fondaparinux OR Bivalirudin OR Dabigatran OR Rivaroxaban OR Apixaban OR Edoxaban OR Betrixaban OR Aspirin OR Clopidogrel OR Prasugrel OR Ticagrelor OR Dipyridamole OR Ticlopidine OR Cangrelor OR Vorapaxar OR Eptifibatide OR Tirofiban OR Atorvastatin OR Simvastatin OR Rosuvastatin OR Pravastatin OR Lovastatin OR Fluvastatin OR Pitavastatin OR Cerivastatin OR Amiodarone OR Sotalol OR Dronedarone OR Flecainide OR Propafenone OR Lidocaine OR Mexiletine OR Quinidine OR Disopyramide OR Procainamide OR Sacubitril/Valsartan OR Spironolactone OR Eplerenone OR Nitroglycerin OR Isosorbide Mononitrate OR Isosorbide Dinitrate OR Erythrityl Tetranitrate OR Pentaerythritol Tetranitrate OR Canagliflozin OR Dapagliflozin OR Empagliflozin OR Sotagliflozin OR semaglutide OR liraglutide OR Dulaglutide OR Exenatide OR tirzepatide OR Bosentan OR Ambrisentan OR Sildenafil OR Tadalafil OR Epoprostenol OR Treprostinil OR Iloprost OR Riociguat OR Selexipag OR Nifedipine OR Diltiazem OR Amlodipine OR Alirocumab OR Evolocumab OR Inclisiran)

The initial search was carried out by two authors (F.C. and A.D.) and all references were reported on an Excel table. After removal duplicates, titles and abstracts of the search results were screened by the same two authors independently. Non-relevant studies were decided at the reviewers’ discretion. At this stage reviewers were overly inclusive to reduce chance of omitting relevant articles. The full text of the remaining results, as well as their reference list, was independently assessed in duplicates by the 2 authors (F.C. and A.D.) for inclusion, based on predetermined inclusion criteria. Any disagreement at this stage was resolved by consensus and by the mediation of a third author (JJ.G.D.). The final list of included studies was decided upon discussion between authors with full agreement required prior to inclusion. A total of 76 articles have been included in the final search.

*Study inclusion criteria*

1) Original observational or prospective studies.

2) The study included a cardiovascular drug for use in patients with cardiovascular disease

3) The study implemented or developed an artificial intelligence algorhytm for diagnostic, prognostic or therapeutic use to impact cardiovascular pharmacotherapy for drugs currently in use

7) English language.

## **Supplementary Chapter 1: Novel Technologies for AI-enabled Remote Blood Pressure Monitoring:**

Out-of-office blood pressure (BP) monitoring has become an indispensable tool in the diagnosis and management of hypertension.^1^ Traditional methods such as home blood pressure monitoring (HBPM) and ambulatory blood pressure monitoring (ABPM) provide valuable insights beyond sporadic office readings, aiding in the identification of various clinical phenotypes like masked hypertension and pathological BP variability, which have significant implications for cardiovascular prognosis.^1^ However, these methods also present limitations, including patient compliance issues, inconvenience, discomfort, and potential for unreliable measurements.^2^ The digital era has witnessed a surge in the development of wearable BP measuring devices, promising more frequent and less intrusive BP monitoring.^3^ These devices leverage different physiological principles and sensor technologies to estimate BP continuously or intermittently in real-world settings:^4^

- Oscillometric Devices: These devices, often resembling smartwatches, utilize the oscillometric technique, similar to traditional automated arm-cuff sphygmomanometers, using a small inflatable cuff or belt on the wrist to detect oscillations in arterial pressure as the cuff deflates.^2^ They have the potential for frequent on-demand or programmed measurements, and minimize discomfort compared to traditional arm cuffs. Yet, limitations related to measurement accuracy secondary to the wrist position relative to the heart, currently limit the accuracy for continuous monitoring only in a sitting position.^5^
- Cuffless Photoplethysmography (PPG)-based Devices: PPG is a non-invasive optical technique that detects changes in blood volume in peripheral tissues using a light source and a photodetector.^6^ Wearable devices, including smartwatches, fitness trackers, and dedicated PPG sensors, can utilize PPG signals to estimate BP. This estimation often relies on pulse transit time, the time it takes for the pressure wave generated by the heartbeat to travel between two arterial sites, which is influenced by BP.^7^ AI algorithms are crucial for calibrating and interpreting PPG signals to derive BP values, and many PPG-based algorithms require individual calibration against a standard cuff-based measurement to establish a baseline. PPG-based devices offer the potential for continuous and unobtrusive BP monitoring without the need for cuff inflation. Yet, the accuracy of PPG-based BP estimation can be influenced by various factors, including motion artifacts, skin tone, temperature changes, and sensor placement.^5^
- Applanation Tonometry-based Devices: This method involves applying a controlled force to a superficial artery to flatten it and measure the pressure within the artery. Tonometry offers a direct, continuous non-invasive measurement of arterial pressure waveform, but maintaining consistent sensor positioning and applied force on the artery can be challenging in wearable devices.^8^
- Other Emerging Technologies: Research is exploring other novel approaches, including BP estimation using electrocardiography signals, ballistocardiography and seismocardiography (measuring whole-body and chest-wall vibrations related to cardiac activity respectively), phonocardiography, ultrasound and even optical imaging of skin blood flow.^5^ Some research also explores integrating chemical sensors in wearables to monitor hypertension-related biochemical parameters. These technologies are generally in earlier stages of development and require further validation.

Despite the promising features, several limitations hinder the widespread clinical adoption of novel remote BP monitoring technologies, as a critical challenge in this field is establishing and validating the accuracy of cuffless BP measuring devices. In this context, movement, body position, and environmental factors can significantly affect the signals acquired by wearable BP monitors, potentially leading to inaccurate readings.^9^ Robust algorithms are needed to mitigate these artifacts in real-world scenarios. In addition, there is still a lack of standardized interpretation for continuous BP monitors data, at difference with traditional methods.^8^ This still represent a barrier for medical treatment adjustment using these information, as established thresholds for cuffless devices are lacking, and evidence for efficacy of medical treatment is hypertension is based on clinical trials using traditional BP methods. Large-scale prospective studies are needed to define clinically relevant BP ranges and their association with cardiovascular events when measured by these novel devices. Hence, the accuracy and reliability of the BP data obtained from wearable devices must be rigorously established before these data can be confidently used to guide pharmacological adjustments remotely.

All these considered, such technology, if critical elements for data accuracy are addressed, enable a transformative impact on hypertension management. Wearable devices can generate extensive BP physiological time-series, providing a richer and more comprehensive understanding of an individual’s BP profile compared to intermittent office or home measurements. This wealth of data, potentially combined with other physiological parameters, such as heart rate, activity levels, glucose levels, and other environmental contextual information, can serve as valuable input for training and refining novel AI algorithms. These could ultimately identify subtle patterns within the physiological series and allow a more precise adjustment of antihypertensive treatment, detecting early signs of suboptimal BP control or adverse drug effects. These also allows future AI-powered platforms, integrated with wearable BP monitors, contextual information, and medical treatment change, to provide patients with personalized feedback on medication efficacy and safety in a closed-loop system. The detailed study of personalized BP trajectories could also predict the individual BP patterns and inform medical treatment change in dose or time. Finally, the digital nature of these data allows a frictionless remote sharing with the physician, to validate and inform treatment management.

## **Supplementary Chapter 2: Novel Technologies for AI-enabled Medication Adherence Tracking:**

Medication nonadherence is a significant global issue contributing to substantial morbidity and mortality, and its impact is particularly pronounced in the management of cardiovascular diseases (CVD). Despite the proven efficacy of evidence-based pharmacotherapies for both primary and secondary prevention of CVD, the benefits are significantly attenuated by patients not taking their medications as prescribed. It is estimated that nearly half of patients do not adhere to their cardiovascular medications, leading to increased risks of cardiovascular events, more frequent hospitalizations, increased healthcare utilization, and higher costs.^10^ Studies have shown that a substantial proportion of individuals with CVD exhibit sub-optimal adherence, with a dramatic increase of non-adherence after the first 6 months of treatment.^11^ Notably, good medication adherence (≥ 80 %) is associated with a 15 % lower risk of CVD events and a 45 % reduction in all-cause mortality compared to non-adherence.^12^ Similarly, adherence to statins and antihypertensive therapy has been linked to significant reductions in CVD risk and mortality.^11^ Poor adherence not only undermines the intended clinical benefits of medications but can also lead to misinterpretations of treatment failure, potentially resulting in unnecessary dose escalations or changes in medication.

Historically, medication adherence has been assessed using various methods, each with its own limitations. Traditional methods include self-reported questionnaires, unstructured inquiries, dispensing records, and pill counts.^10^ Self-reports, while easy to administer, are prone to recall bias and social desirability bias, often leading to overestimation of adherence. Dispensing records from pharmacies can provide insights into medication refills but do not confirm actual ingestion by the patient. Pill counts, where patients return unused medication, assume that all missing pills were taken as prescribed, which may not always be the case, and can be influenced by intentional disposal of medication.^13^ Pharmacologic measurement of drug or metabolite levels in bodily fluids is a direct measure but often requires specialized laboratory support, making it infeasible for broad adoption in routine clinical practice. These traditional methods often provide complementary rather than gold-standard information, making it challenging to accurately monitor and understand patterns of medication-taking behavior.^14^

To overcome the limitations of traditional methods, novel digital health technologies have emerged as potential tools for more accurately monitoring and improving medication adherence in patients with CVD. These technologies encompass a wide range of devices and systems, including electronic pill bottles or boxes, ingestible sensors, electronic medication management systems, blister pack technology, smart medicine cabinets, wearable devices, smartphone applications, telemedicine platforms, and AI-powered systems. Electronic pill bottles, such as the Medication Event Monitoring System (MEMS), have a microchip in the cap that records the date and time each time the bottle is opened, providing objective data on medication access .^15,16^ Ingestible sensor systems represent a novel, direct method of measuring adherence by detecting when a medication is ingested. These sensors, often integrated into a pill or co-packaged with medication, transmit a signal upon contact with stomach fluids, providing a near–real-time record of ingestion.^17^

Smartphone applications (apps) offer various functionalities to support medication adherence, such as reminders, educational information, tracking of medication intake, and integration with other health data. Systematic reviews and meta-analyses have explored the effectiveness of these apps in improving medication adherence in CVD patients, with some trials showing statistically significant improvements compared to control groups.^14^ However, the evidence remains somewhat inconsistent, and the effectiveness may depend on the specific features of the app, the duration of use, and the characteristics of the patient population. Wearable devices, such as smartwatches and activity trackers, can also play a role in adherence by providing reminders and by monitoring physiological parameters that may be linked to medication adherence and treatment outcomes, such as blood pressure and physical activity. Smart medicine cabinets aim to measure access and ingestion of medications through electronic sensors and can provide behavioural prompts. Telemedicine platforms facilitate remote monitoring of medication adherence through patient-reported data and video consultations, allowing for personalized advice and support from healthcare providers.

The integration of these adherence monitoring technologies with AI holds significant promise for enhancing medication adherence and ultimately improving outcomes in cardiovascular pharmacotherapy. AI algorithms can analyze the data generated by these technologies, such as ingestion times from digital pills, opening events from electronic pill bottles, and patient interactions with smartphone apps, to identify patterns of adherence and non-adherence. Machine-learning techniques can be used to predict which patients are at higher risk of non-adherence based on various factors, including their medication history, demographics, and behavioural data. AI-powered systems can then deliver personalized interventions tailored to the individual patient’s needs and barriers to adherence. For example, AI could analyse patterns of missed doses and send timely, adaptive reminders via smartphone apps or other connected devices. AI-driven virtual coaches could provide motivational support, address concerns about side effects, and offer strategies for integrating medication schedules into daily routines.

Finally, novel technologies for extended reality, such as smart glasses, have the potential to be extremely useful for medication adherence by leveraging embedded multimodal AI models for contextual reality interpretation.^18^ Such systems could potentially act as a real-time reminder and address the question of whether the dosing of the medication was actually ingested (“did I already take the pill?”). They could also offer advanced contextual understanding (“did I take pill A or B?”, “was the dosage correct?”) through features like object recognition. Furthermore, smart glasses could enable effective medication-adherence tracking (is the patient adherent to the prescribed treatment plan over the longer term?) by potentially leveraging continuous video recording and contextual interpretation of observed actions by a multimodal AI model. This approach could potentially solve multiple problems associated with current adherence-tracking devices, with limited intrusion and cost compared to methods requiring active patient input or dedicated devices.^19^

Despite the opportunities, there are current limitations to the widespread adoption and effectiveness of these technologies and their integration with AI. The accuracy of some monitoring technologies in real-world settings may be affected by various factors, including patient adherence to the technology itself. Technological barriers such as limited access to smartphones and the internet, as well as limited digital literacy, can create inequalities in the use and benefits of digital health interventions. Ethical concerns surrounding data privacy and security are also paramount when collecting and analyzing sensitive medication-adherence data. The clinical effectiveness and cost-effectiveness of many of these interventions, particularly in the long term and across diverse patient populations, require further rigorous evaluation through large-scale clinical trials. It remains challenging to determine which specific features or combinations of features in digital health interventions are most effective for improving adherence. Looking to the future, the field of medication-adherence monitoring and intervention in cardiovascular pharmacotherapy is poised for significant advancements. Continued innovation in sensor technology, wearable devices, and AI algorithms will likely lead to more accurate, unobtrusive, and personalized approaches to supporting medication adherence, but should be supported by high-quality evidence.^10,20^

**Supplementary References:**

1. Stergiou GS, Palatini P, Parati G, O’Brien E, Januszewicz A, Lurbe E, et al. 2021 European Society of Hypertension practice guidelines for office and out-of-office blood pressure measurement. Journal of Hypertension 2021;39:1293–1302.

2. Kario K. Management of Hypertension in the Digital Era: Small Wearable Monitoring Devices for Remote Blood Pressure Monitoring. Hypertension 2020;76:640–650.

3. Sinou N, Sinou N, Koutroulakis S, Filippou D. The Role of Wearable Devices in Blood Pressure Monitoring and Hypertension Management: A Systematic Review. Cureus 2024.

4. Juyal A, Bisht S, Singh MF. Smart solutions in hypertension diagnosis and management: a deep dive into artificial intelligence and modern wearables for blood pressure monitoring. Blood Pressure Monitoring 2024;29:260–271.

5. Konstantinidis D, Iliakis P, Tatakis F, Thomopoulos K, Dimitriadis K, Tousoulis D, et al.. Wearable blood pressure measurement devices and new approaches in hypertension management: the digital era. J Hum Hypertens 2022;36:945–951.

6. Elgendi M, Fletcher R, Liang Y, Howard N, Lovell NH, Abbott D, et al.. The use of photoplethysmography for assessing hypertension. npj Digit Med 2019;2:60.

7. Liang Y, Abbott D, Howard N, Lim K, Ward R, Elgendi M. How Effective Is Pulse Arrival Time for Evaluating Blood Pressure? Challenges and Recommendations from a Study Using the MIMIC Database. JCM 2019;8:337.

8. Stergiou GS, Avolio AP, Palatini P, Kyriakoulis KG, Schutte AE, Mieke S, et al. European Society of Hypertension recommendations for the validation of cuffless blood pressure measuring devices: European Society of Hypertension Working Group on Blood Pressure Monitoring and Cardiovascular Variability. Journal of Hypertension 2023;41:2074–2087.

9. Armoundas AA, Ahmad FS, Attia ZI, Doudesis D, Khera R, Kyriakoulis KG, et al. Controversy in Hypertension: Pro-Side of the Argument Using Artificial Intelligence for Hypertension Diagnosis and Management. Hypertension 2025:HYPERTENSIONAHA.124.22349.

10. Valgimigli M, Garcia-Garcia HM, Vrijens B, Vranckx P, McFadden EP, Costa F, et al. Standardized classification and framework for reporting, interpreting, and analysing medication non-adherence in cardiovascular clinical trials: a consensus report from the Non-adherence Academic Research Consortium (NARC). European Heart Journal 2019;40:2070–2085.

11. Jackevicius CA. Adherence With Statin Therapy in Elderly Patients With and Without Acute Coronary Syndromes. JAMA 2002;288:462.

12. Islam SMS, Chow CK, Daryabeygikhotbehsara R, Subedi N, Rawstorn J, Tegegne T, et al. Wearable cuffless blood pressure monitoring devices: a systematic review and meta-analysis. European Heart Journal - Digital Health 2022;3:323–337.

13. Pocock SJ, Abdalla M. The hope and the hazards of using compliance data in randomized controlled trials. Statist Med 1998;17:303–317.

14. Al-Arkee S, Mason J, Lane DA, Fabritz L, Chua W, Haque MS, et al. Mobile Apps to Improve Medication Adherence in Cardiovascular Disease: Systematic Review and Meta-analysis. J Med Internet Res 2021;23:e24190.

15. Blaschke TF, Osterberg L, Vrijens B, Urquhart J. Adherence to Medications: Insights Arising from Studies on the Unreliable Link Between Prescribed and Actual Drug Dosing Histories. Annu Rev Pharmacol Toxicol 2012;52:275–301.

16. El Alili M, Vrijens B, Demonceau J, Evers SM, Hiligsmann M. A scoping review of studies comparing the medication event monitoring system (MEMS) with alternative methods for measuring medication adherence. Brit J Clinical Pharma 2016;82:268–279.

17. Chai PR, Vaz C, Goodman GR, Albrechta H, Huang H, Rosen RK, et al. Ingestible electronic sensors to measure instantaneous medication adherence: A narrative review. DIGITAL HEALTH 2022;8:205520762210831.

18. Taghian A, Abd El‐Malek AH, Sayed MS, Abo‐Zahhad M. Developing an integrated medication adherence system: Exploring the potential of i‐Ware’s augmented reality goggles and mobile application. IET Smart Cities 2023;5:230–242.

19. Lee H, Youm S. Development of a Wearable Camera and AI Algorithm for Medication Behavior Recognition. Sensors 2021;21:3594.

20. Etminani K, Göransson C, Galozy A, Norell Pejner M, Nowaczyk S. Improving Medication Adherence Through Adaptive Digital Interventions (iMedA) in Patients With Hypertension: Protocol for an Interrupted Time Series Study. JMIR Res Protoc 2021;10:e24494.

**Supplementary Table 1. Glossary of Terms**

| **Term** | **Explanation** |
| --- | --- |
| **Machine Learning** | A branch of artificial intelligence that focuses on developing computer systems capable of learning and adapting automatically without being explicitly programmed. These systems use algorithms and statistical models to identify patterns in data, draw inferences, and improve their performance on specific tasks as they are exposed to more data. |
| **(Artificial) Neural Network** | Computational models inspired by biological neural networks, consisting of interconnected nodes (neurons) that process and transmit information. These nodes adjust their connections (weights) during learning, with each node's output determined by an activation function applied to the sum of its inputs, allowing the network to adapt to specific tasks. |
| **Deep Neural Network** | A type of artificial neural network with multiple layers between the input and output, known as 'hidden layers.' These layers allow the network to model complex patterns in data. |
| **Deep Learning** | A type of machine learning that uses multi-layered neural networks to learn and extract features from large, complex datasets. |
| **Convolutional Neural Network** | A specialized type of artificial neural network designed for processing structured grid-like data, such as images. It uses convolutional layers to automatically detect and learn spatial patterns like edges, textures, or shapes, making them highly effective for image recognition and classification. |
| **Decision Trees** | A machine learning algorithm that splits data into branches based on feature values, leading to decisions at each node. The final output is based on the decisions made along the branches, making it useful for classification and regression tasks. |
| **Random Forest** | A machine learning algorithm that builds multiple decision trees independently from random subsets of data and features. The final prediction is based on averaging (for regression) or majority voting (for classification). |
| **Causal Forest** | An extension of the random forest method for causal inference. |
| **Gradient Boosting** | A machine learning technique that builds models sequentially, where each new model aims to correct the errors of the previous one. It is particularly effective in reducing bias and improving predictive accuracy. |
| **Extreme Gradient Boosting -XGBoost** | A machine learning algorithm that uses gradient boosting, combining decision trees sequentially. Each new tree corrects errors made by the previous trees, leading to a more accurate model over time. |
| **Variational Autoencoder** | A type of neural network with an encoder that compresses input data into a simpler representation and a decoder that reconstructs the original data from this compressed form. |
| **Hierarchical Clustering** | A clustering method that builds a tree-like structure (dendrogram) to represent data groupings based on their similarities. It can be agglomerative (starting with individual points and merging) or divisive (starting with all points and splitting). This method does not require specifying the number of clusters in advance, allowing flexible exploration of data at different levels. |
| **Support Vector Methods** | A group of machine learning algorithms, including support vector machines (for classification) and support vector regression (for regression), that use support vectors—key data points in the dataset. During training, these methods select the most important data points (support vectors) and adjust the boundary (for classification) or line (for regression) to achieve the best separation between groups or the most accurate predictions for numerical values. |
| **Natural Language Processing** | A field of AI that focuses on the interaction between computers and human language. It is used to analyze, understand, and generate natural language, often applied to extract information from unstructured text data, like medical records. |
| **Quantitative Structure-Activity Relationship** | A method in computational chemistry used to analyze the relationship between a molecule’s chemical structure and its biological activity. QSAR relies on supervised machine learning and statistical models that use manually defined descriptors, which capture predefined molecular properties such as size, shape, and electronic distribution. By identifying patterns in known compounds, QSAR can predict the effects of new molecules, aiding in drug discovery and chemical safety assessments. |
| **Footprints/Fingerprints** | Small molecular fragments that can serve as building blocks for drug design of drug classes |

**Supplementary Table 2: Studies for artificial intelligence in cardiovascular pharmacotherapy**

| **Study Title** | **Year** | **Clinical Area** | **Study Type** | **Overall Population Size** | **Reference** | **Validation Type** | **Model Type** | **Model Category** | **Model Features** | **Model Outcomes** | **Model Performance (Internal)** | **Model Performance (External)** |
| --- | --- | --- | --- | --- | --- | --- | --- | --- | --- | --- | --- | --- |
| **Noninvasive assessment of dofetilide plasma concentration using a deep learning (neural network) analysis of the surface electrocardiogram: A proof of concept study.** | 2018 | Arrhythmia | Retrospective | 42 | 10.1371/journal.pone.0201059 | Internal | Neural network | Neural Networks | Clinical variables, demographic, medication and electrocardiogram parameters | Dofetilide plasma concentration | Model prediction correlation with observed drug levels (r=0.85) | N.A. |
| **Applications of machine learning in decision analysis for dose management for dofetilide.** | 2019 | Arrhythmia | Retrospective | 354 | 10.1371/journal.pone.0227324 | Internal | Tabular reinforcement-learning model | Other | Clinical variables, demographic, medication, echocardiogram and electrocardiogram parameters. | Dose management for dofetilide | Model accuracy 96,1% | N.A. |
| **Deep learning analysis of electrocardiogram for risk prediction of drug-induced arrhythmias and diagnosis of long QT syndrome** | 2021 | Arrhythmia | Retrospective | 1564 | 10.1093/eurheartj/ehab588 | External | Convolutional neural network | Neural Networks | Clinical variables, demographic, medication and electrocardiograms. | Drug-induced Torsade de Pointes | Exposure to sotalol (ROC-AUC= 0.98 vs. 0.72, P ≤ 0.001). The presence and type of cLQTS vs. healthy controls, particularly for cLQT2 (AUC-ROC = 0.9) | N.A. |
| **Evaluation of Quantitative Decision-Making for Rhythm Management of Atrial Fibrillation Using Tabular Q-Learning** | 2023 | Arrhythmia | Retrospective | 52547 | 10.1161/JAHA.122.028483 | Internal | Tabular Q-learning (batch gradient descent) | Other | Clinical variables | Optimal initial rhythm-management strategy | Total deaths (8.5% vs. 22.4%, OR=0.32, P=0.02). | Deaths (18.5% vs. 23.0%, OR, 0.76, P=0.044) and longer time to event (1325.9±938.5 days vs. 1225.6±907.8 days, P=0.046), with larger overall reward (−0.210±1.14 versus −0.43±2.20, P=0.004) |
| **Explainable Machine Learning Techniques To Predict Amiodarone-Induced Thyroid Dysfunction Risk: Multicenter, Retrospective Study With External Validation** | 2023 | Arrhythmia | Retrospective | 6497 | 10.2196/43734 | External | XGboost | Tree-based / Ensemble Methods | Clinical variables, demographic, medication and laboratory parameters. | Amiodarone-induced thyroid dysfunction | Accuracy 0.923; Precision 0.632, recall 0.756, F1-score 0.688, G-mean 0.845, AUPRC 0.751, and AUROC 0.934 | Among patients  who developed AITD, 71.3% had a predicted high risk.  Among the  remaining non-AITD patients, 95.8% had a  predicted risk lower than the optimal threshold and were thus true negatives estimated by the model. |
| **Prediction of high on-treatment platelet reactivity in clopidogrel-treated patients with acute coronary syndromes** | 2018 | Coronary Artery Disease | Retrospective | 603 | 10.1016/j.ijcard.2017.03.074 | Internal | ANN | Neural Networks | Clinical variables, demographic and genetics. | Platelet reactivity in clopidogrel-treated patients | Accuracy 63% (95% CI 59-66) | N.A. |
| **Machine learning versus traditional risk stratification methods in acute coronary syndrome: a pooled randomized clinical trial analysis** | 2020 | Coronary Artery Disease | Retrospective | 24178 | 10.1007/s11239-019-01940-8 | Internal | Super learner ensemble algorithm | Tree-based / Ensemble Methods | Clinical variables, demographic, laboratory and medication. | MACE and bleeding in patients with ACS | MACE  c-statistic 0.734 vs. logistic regression 0.714.  Bleeding c-statistic 0.670 vs. logistic regression 0.671 | N.A. |
| **Machine learning-based prediction of adverse events following an acute coronary syndrome (PRAISE): a modelling study of pooled datasets** | 2021 | Coronary Artery Disease | Retrospective | 19862 | 10.1016/S0140-6736(20)32519-8 | External | Adaptive boosting, naive Bayes, K-nearest neighbours, and random forest | Tree-based / Ensemble Methods | Clinical variables, demographic, laboratory, procedural variables and medication. | All-cause death, recurrent acute myocardial infarction, and major bleeding after ACS | AUC 0.82 (95% CI 0.78-0.85) for 1-year all-cause death; AUC 0.74 (95% CI 0.70-0.78) 1-year myocardial infarction; AUC 0.70 (95% CI 0.66-0.75) for 1-year major bleeding | AUC 0.92 (95% CI 0.90-0.93) for 1-year all-cause death; AUC 0.81 (95% CI 0.76-0.85) for 1-year myocardial infarction; AUC  0.86 (95% CI 0.82-0.89) for 1-year major bleeding |
| **Model-Informed Dosing Regimen of Ticagrelor in Chinese Patients With Acute Coronary Syndrome** | 2023 | Coronary Artery Disease | Retrospective | 178 | 10.1002/cpt.3048 | Internal | Decision Tree, Linear Discriminant Analysis, Support Vector Machine, Random Forest, K-nearestNeighbor, Extreme Gradient Boosting, and NaiveBayes algorithms | Mixed | Clinical, vital signs, drinking status and treatment. | Bleeding risk | RF model F1 score of 0.73 and ROC-AUC of 0.81. Logistic Regression AUC 0.74; DT AUC 0.69; LDA AUC 0.75; SVM ACU 0.79; KNN AUC 0.81; XGBoost 0.69; NB AUC 0.71 | N.A. |
| **A novel machine learning model to predict high on-treatment platelet reactivity on clopidogrel in Asian patients after percutaneous coronary intervention** | 2024 | Coronary Artery Disease | Retrospective | 507 | 10.1007/s11096-023-01638-1 | Internal | XGBoost | Tree-based / Ensemble Methods | Clinical, demographics, medication, laboratory, stent-related characteristics, and genetics | High on-treatment platelet reactivity in patients taking clopidogrel | AUC of 0.82, precision of 0.80, recall of 0.44, F1 score of 0.57, accuracy of 0.87 | N.A. |
| **Myocardial infarction in type 2 diabetes using sodium-glucose co-transporter-2 inhibitors, dipeptidyl peptidase-4 inhibitors or glucagon-like peptide-1 receptor agonists: proportional hazards analysis by deep neural network based machine learning** | 2020 | Diabetes | Retrospective | 199116 | 10.1080/03007995.2019.1706043 | Internal | Deep neural network | Neural Networks | Clinical variables, demographic, laboratory and medication. | Incidence of cardiovascular disease among patients with type 2 diabetes using antidiabetic drugs from three classes | Significantly lower risk of MI with SGLT-2is or GLP-1RAs than DPP-4is (HR  0.81, 95% CI:  0.72-0.91, p = .0004 vs. 0.63, 0.56-0.72, p < 0.0001). MI risk was also significantly lower with GLP-1RAs than SGLT-2is (0.77, 0.66-0.90, p = 0.001) | N.A. |
| **Development and Validation of a Machine Learning Model to Predict Near-Term Risk of Iatrogenic Hypoglycemia in Hospitalized Patients.** | 2021 | Diabetes | Retrospective | 35147 | 10.1001/jamanetworkopen.2020.30913 | External | Stochastic gradient boosting | Tree-based / Ensemble Methods | Clinical variables, vital signs, demographic, laboratory and medication. | Iatrogenic hypoglycemia within 24 hours after each blood glucose measurement | C statistic of 0.90 (95% CI, 0.89-0.90), a positive predictive value of 0.09 (95% CI 0.08-0.09), a positive likelihood ratio of 4.67 (95% CI 4.59-4.74), a negative predictive value of 1.00 (95% CI, 1.00-1.00), and a negative likelihood ratio of 0.22 (95% CI, 0.21-0.23) | C statistics ranging from 0.86 to 0.88, positive predictive values ranging from 0.12 to 0.13, negative predictive values of 0.99, positive likelihood ratios ranging from 3.09 to 3.89, and negative likelihood ratios ranging from 0.23 to 0.25. |
| **Using machine learning to identify diabetes patients with canagliflozin prescriptions at high-risk of lower extremity amputation using real-world data.** | 2021 | Diabetes | Retrospective | 13904 | 10.1002/pds.5206 | Internal | Elastic net, least absolute shrinkage and selection operator, gradient boosting machine and random forests | Tree-based / Ensemble Methods | Clinical variables, demographic and medication. | Lower extremity amputation among diabetes patients treated with canagliflozin | LASSO produced the best prediction, yielding a C-statistic of 0.81 (95% CI 0.76, 0.86); gradient boosting machine (C-statistic 0.79, 95%CI  0.73, 0.84); elastic net (C-statistic 0.77, 95%CI:  0.73, 0.83) or random forest (C statistic 0.65, 95% CI:  0.58, 0.72). At sensitivity level of 90%, LASSO showed a better performance compared to gradient boosting machine, having higher specificity (58.30% vs. 49.87%) | N.A. |
| **Phenomapping-Derived Tool to Individualize the Effect of Canagliflozin on Cardiovascular Risk in Type 2 Diabetes.** | 2022 | Diabetes | Retrospective | 10135 | 10.2337/dc21-1765 | External | Extreme gradient boosting | Tree-based / Ensemble Methods | Clinical variables, demographic, laboratory and electrocardiogram parameters. | Atherosclerotic cardiovascular disease benefit of canagliflozin in type 2 diabetes | Root mean square error of 0.46. | Individuals with the highest predicted  benefit had a lower risk when treated with canagliflozin compared with placebo (Adjusted HR 0.60 , 95% CI 0.41–0.89) at difference with the general effect in the external validation cohort (Adjusted HR 0.99, 95% CI 0.76–1.30, ; P interaction = 0.04) |
| **Heterogeneous treatment effects of intensive glycemic control on major adverse cardiovascular events in the ACCORD and VADT trials: a machine-learning analysis.** | 2022 | Diabetes | Retrospective | 12042 | 10.1186/s12933-022-01496-7 | Internal | Causal forests machine learning analysis | Tree-based / Ensemble Methods | Clinical variables, demographic, laboratory and medication. | Treatment effects of intensive glycemic control in type 2 diabetes patients on MACE | The algorhytm was able to identify low (- 4.2% [95% CI - 8.1, - 1.0]), intermediate (- 5.1% [95% CI - 8.7, - 1.5]), and high (- 4.3% [95% CI - 7.7, - 1.0]) MACE risk patients | N.A. |
| **Exploring metformin monotherapy response in Type-2 diabetes: Computational insights through clinical, genomic, and proteomic markers using machine learning algorithms.** | 2024 | Diabetes | Retrospective | 62 | 10.1016/j.compbiomed.2024.108106 | Internal | Support Vector Machine (SVM), Naive Bayes (NB), K-Nearest Neighbour (K-NN), Logistic Regression (LR) and Random Forest (RF) | Mixed | Clinical variables, demographic, genomics and proteomics. | Metformin response | When classifying control and responder groups the Support Vector Machine model shows maximal sensitivity of 76%, specificity of 76%, a MCC score of 0.48 and an AUC of 0.73. The performance of k-NN model in classifying control and non-responder groups shows maximal sensitivity of 83%, specificity of 82%, a MCC score of 0.63 and an AUC of 0.81. When classifying control and treatment to metformin monotherapy groups, the Naïve Bayes model shows maximal sensitivity of 86%, specificity of 85%, a MCC score of 0.55 and an AUC of 0.72 | N.A. |
| **Identifying Drug-Drug Interactions by Data Mining: A Pilot Study of Warfarin-Associated Drug Interactions** | 2016 | Drug adherence/adverse effects | Retrospective | 13695 | 10.1161/CIRCOUTCOMES.116.003055 | Internal | Random forest | Tree-based / Ensemble Methods | Clinical variables, demographic, laboratory and medication. | Discover drug-drug interactions | AUC for each of the 3 classes (nonevent; increasing INR; and decreasing INR) were between 0.66 and 0.72. Sensitivity and Specificity (Non events 0.6, 0.78), (High INR 0.56, 0.81), (Low INR 0.62, 0.79) | N.A. |
| **Using Artificial Intelligence to Reduce the Risk of Nonadherence in Patients on Anticoagulation Therapy** | 2017 | Drug adherence/adverse effects | Prospective | 28 | 10.1161/STROKEAHA.116.016281 | Internal | – | Other | Clinical variables, demographic, laboratory and medication. | Medication adherence | Mean cumulative adherence based on pill count was 97.2% (4.4%) for the AI platform group and 90.6% (5.8%) for the control group. Adherence was 100% (15 of 15) and 50% (6 of 12) in the intervention and control groups | N.A. |
| **Behavioral Nudges as Patient Decision Support for Medication Adherence: The ENCOURAGE Randomized Controlled Trial.** | 2022 | Drug adherence/adverse effects | Prospective | 182 | 10.1016/j.ahj.2021.11.001 | External | Behavioral nudges driven by artificial intelligence | Other | Clinical, demographic, laboratory and medication. | Medication adherence | Proportion of days covered (0.742±0.318) compared to controls ( 0.639±0.358, P = 0.042). Adherent subjects  (66.3% vs controls:  50.5%, P = 0.036) | N.A. |
| **Artificial Intelligent Context-Aware Machine-Learning Tool to Detect Adverse Drug Events from Social Media Platforms** | 2022 | Drug adherence/adverse effects | Prospective |  | 10.1007/s13181-022-00906-2 | Internal | Natural language processing (aTarantula) -  FastText embeddings | NLP / Text-based Models | Patient-centric online discussion forums | Adverse drug events | Sensitivity of 84.2% and a specificity of 98% | N.A. |
| **Robust Performance of Potentially Functional SNPs in Machine Learning Models for the Prediction of Atorvastatin-Induced Myalgia** | 2021 | Dyslipidemia | Retrospective | 183 | 10.3389/fphar.2021.605764 | Internal | Logistic Regression, Elastic Net, Random Forest, Boosted tree, Neural Network, Support vector machine | Mixed | Clinical variables, demographic, genetics and medication. | Myalgia | A combination of 15 single nucleotide polymorphisms predict myalgia with good predictive performance (AUC >0.9) | N.A. |
| **Producing personalized statin treatment plans to optimize clinical outcomes using big data and machine learning.** | 2022 | Dyslipidemia | Retrospective | 38214 | 10.1016/j.jbi.2022.104029 | Internal | Artificial Neural Network | Neural Networks | Clinical variables, demographic, laboratory and medication. | Otimal treatment plan, by means of statin type and dosage, to prevent or minimize statin-associated adverse symptoms | NN model showed ROC-AUC 65.04, PR-AUC 63.86, Accuracy 61.38, Recall 55.27 and Precision 62.88 | N.A. |
| **Using deep learning-based natural language processing to identify reasons for statin nonuse in patients with atherosclerotic cardiovascular disease.** | 2022 | Dyslipidemia | Retrospective | 56530 | 10.1038/s43856-022-00157-w | Internal | deep learning-based natural language processing (NLP) | Neural Networks | Clinical variables, demographic and medication. | Reasons for statin nonuse in patients with atherosclerotic cardiovascular disease | Model ability to predict statin nonadherence (AUC:  0.94, 95% CI 0.93-0.96). Model ability to predict reasons for nonuse (AUC:  0.88, 95% CI 0.86-0.91) | N.A. |
| **Machine-learning-based analysis of the sensitivity and specificity on lipid-lowering effect of one-month-administered statins** | 2023 | Dyslipidemia | Retrospective | 1013 | 10.1097/MD.0000000000033139 | Internal | Random forest | Tree-based / Ensemble Methods | Clinical variables, demographic and laboratory parameters. | Response to atorvastatin | LDL:  training accuracy  100%; the correct rate of test, 91.80%; sensitivity, 86.86%; specificity 94.83%,  AUC = 0.97. Triglyceride:  training accuracy  100%; the correct rate of test, 71.7%; sensitivity,71.21%; specificity, 73.46%, AUC = 0.80. Total cholesterol:  the training accuracy 100%; the correct rate of test, 94.90%; the sensitivity, 94.38%;  specificity, 96.55%, AUC =0.98. HDL:  training accuracy of | N.A. |
| **Machine Learning Applied to Cholesterol-Lowering Pharmacotherapy: Proof-of-Concept in High-Risk Patients Treated in Primary Care.** | 2023 | Dyslipidemia | Retrospective | 5630 | 10.1089/met.2023.0009 | Internal | Neural network. Natural language processing and therapy identification algorithms | Neural Networks | Clinical variables, demographic, laboratory and medication. | Identifing suboptimal lipid-lowering prescribing patterns, and identifying high-risk patients who could benefit from more intensive therapy. | The model recommended either increasing the dose of statin, adding complementary cholesterol-lowering medication, or obtaining an expert lipid opinion adding complementary cholesterol-lowering medication | N.A. |
| **Machine Learning Methods Improve Prognostication, Identify Clinically Distinct Phenotypes, and Detect Heterogeneity in Response to Therapy in a Large Cohort of Heart Failure Patients** | 2018 | Heart Failure | Retrospective | 44886 | 10.1161/JAHA.117.008081 | Internal | Random forest | Tree-based / Ensemble Methods | Clinical variables, demographic, laboratory, medication and echocardiogram. | Prognostication of outcomes, identify distinct patient phenotypes, and detect heterogeneity in treatment response | Discriminate individual risk AUC=0.83. Survival (C-statistic=0.83). Left ventricular ejection fraction did not (C-statistic=0.52). Differences in outcomes per cluster Cluster 1 (hazard ratio [Hazard Ratio (HR)] 3.31, 95% confidence interval [Confidence Interval (CI)], 3.04–3.59), Cluster 3 (Hazard Ratio (HR) 4.52, 95% Confidence Interval (CI), 4.18–4.89), and Cluster 4 (Hazard Ratio (HR) 1.19, 95% Confidence Interval (CI), 1.07–1.33) | N.A. |
| **Novel endotypes in heart failure: effects on guideline-directed medical therapy.** | 2018 | Heart Failure | Retrospective | 2615 | 10.1093/eurheartj/ehy712 | External | Principal component analysis, Unsupervised cluster analysis | Other | Clinical variables, demographic, medication and biomarkers. | Determine subtypes of patients with heart failure with a distinct clinical profile and treatment response | The identified endotype 4 had more severe symptoms and signs of Heart Failure (HF), higher NT-proBNP levels and highest risk for all-cause mortality or hospitalization for Heart Failure (HF) [Hazard Ratio (HR) 1.4; 95% confidence interval (Confidence Interval (CI)) 1.1-1.8]. Endotypes 2, 3, and 5 were better uptitrated to target doses of beta-blockers (P < 0.02 for all). Endotype 2 (Hazard Ratio (HR) 1.29; 95% Confidence Interval (CI) 1.10-1.42) experienced possible harm from uptitration of beta-blockers (Pinteraction for all <0.001) | External validation confirmed, similar to the development cohorts, worst outcomes in endotype 4 identified patients and best outcomes in endotype 1 identified patients. |
| **Redefining β-blocker response in heart failure patients with sinus rhythm and atrial fibrillation: a machine learning cluster analysis** | 2021 | Heart Failure | Retrospective | 15659 | 10.1016/S0140-6736(21)01638-X | Internal | Hierarchical clustering. Bootstrapping | Other | Clinical variables, demographic, medication, laboratory, echocardiograms and electrocardiograms. | Prognostic response from β blockers in patients with heart failure and reduced LVEF | In sinus rythm, most clusters demonstrated a consistent overall mortality benefit from β blockers, with odds ratios (ORs) ranging from 0·54 to 0·74 (p<0,001). Robustness of clustering in sinus rhythm (mean Jaccard score 0·575 [SD 0·103], compared with 0·121 [SD 0·005] | N.A. |
| **Treatment response to spironolactone in patients with heart failure with preserved ejection fraction: a machine learning-based analysis of two randomized controlled trials** | 2023 | Heart Failure | Retrospective | 947 | 10.1016/j.ebiom.2023.104795 | External | XGboost | Tree-based / Ensemble Methods | Clinical, laboratory and ecocardiographic parameters | Response to spironolactone by echocardiographic improvement in E/e' pattern. | AUC 0.87 (in discriminating between patients responders) | Responders (HR 0.42, 95% CI 0.22, 0.78; p = 0.008) vs in the non-responder group (HR 0.88, 95% CI 0.59, 1.31; p = 0.52 |
| **Prediction of Left Ventricular Ejection Fraction Change Following Treatment With Sacubitril/Valsartan** | 2023 | Heart Failure | Retrospective | 416 | 10.1016/j.jchf.2022.09.009 | Internal | XGBoost | Tree-based / Ensemble Methods | Clinical variables, demographic, medication, echocardiogram and laboratory parameters. | LVEF change after Sacubitril/Valsartan therapy | AUC:  0.86 | N.A. |
| **A Machine Learning Model to Predict Diuretic Resistance.** | 2023 | Heart Failure | Retrospective | 16132 | 10.34067/KID.0005562022 | External | XGBoost | Tree-based / Ensemble Methods | Clinical variables, vital signs, demographic, laboratory and medication. | Diuretic resistance | Model discrimination in the training set AUC 0.77 and testing set AUC 0.77. Specificity of 66% in the training set and 66.9% in the testing set. | AUC 0,72. Accuracy 60%. Specificity was 92%, yielding a positive likelihood ratio of 3.46. C-statistic 0.69. |
| **Clinical Value of Predicting Individual Treatment Effects for Intensive Blood Pressure Therapy** | 2019 | Hypertension | Retrospective | 14094 | 10.1161/CIRCOUTCOMES.118.005010 | Internal | X-learner | Tree-based / Ensemble Methods | Clinical variables, demographic, laboratory and medication. | Benefit from intensive blood pressure therapy and predict heterogeneous treatment effects | Logistic regression had lower C statistic for benefit than the X-learner (0.51 [95% Confidence Interval (CI), 0.49-0.53] versus 0.60 [95% Confidence Interval (CI), 0.58-0.63], respectively). Individualized therapy in LR 1065.47 days (95% Confidence Interval (CI), 1061.04-1069.35) vs X-learner's 1068.71 days (95% Confidence Interval (CI), 1065.42-1072.08). ARR LR vs X-learner´s of 0.73 [95% Confidence Interval (CI), 0.30-1.14] versus 1.06 [95% Confidence Interval (CI), 0.74-1.32]. | N.A. |
| **Predicting Optimal Hypertension Treatment Pathways Using Recurrent Neural Networks** | 2020 | Hypertension | Retrospective | 245499 | 10.1016/j.ijmedinf.2020.104122 | Internal | ANN | Neural Networks | Clinical variables, demographic, medication. | Identify optimal hypertension treatment pathways | BP (<140 mmHg), diastolic BP (<90 mmHg), and both systolic BP and diastolic BP (<140/90 mmHg), F1-scores were 0.928, 0.960, and 0.913, respectively | N.A. |
| **Characterizing the critical features when personalizing antihypertensive drugs using spectrum analysis and machine learning methods** | 2020 | Hypertension | Retrospective | 19570 | 10.1016/j.artmed.2020.101841 | Internal | Least absolute shrinkage and selection operator(Lasso) model, mean decrease impurity model, stability selection model, recursive feature elimination model, and the ensemble model. | Tree-based / Ensemble Methods | Demographic and laboratory | Matching patient’s attributes with drug-related features | Differentials for five drug group evaluated through statistical and ensembled models | N.A. |
| **Personalized prescription of ACEI/ARBs for hypertensive COVID-19 patients** | 2021 | Hypertension | Retrospective | 3643 | 10.1007/s10729-021-09545-5 | External | RF, CART, OCT,  XGBoost | Tree-based / Ensemble Methods | Clinical variables, demographic, laboratory and medication. | Personalize prescription of ACEIs and ARBs | AUC:  XGBoost Training 0.909; Testing 0.802. RF Training 0.886; Testing 0.834. | AUC XGBoost 0.768. AUC RF 0.77 |
| **Individualising intensive systolic blood pressure reduction in hypertension using computational trial phenomaps and machine learning: a post-hoc analysis of randomised clinical trials** | 2022 | Hypertension | Retrospective | 14094 | 10.1016/S2589-7500(22)00170-4 | External | XGBoost | Tree-based / Ensemble Methods | Clinical, demographic, laboratory, medication and electrocardiograms parameters. | Benefit of intensive control of systolic blood pressure on MACE | HR for MACE was 0.63 (IQR 0.53-0.78). (R²=0.47) | HR for time-to-first MACE of 0.70 (95% CI 0.55–0.90) in high responders versus 1.05 (95% CI 0.84–1.32) in low responders. |
| **Prediction and evaluation of combination pharmacotherapy using natural language processing, machine learning and patient electronic health records** | 2022 | Hypertension | Retrospective | – | 10.1016/j.jbi.2022.104164 | External | Novel phenome-driven drug discovery system (TuSDC). Natural language processing | NLP / Text-based Models | Clinical variables and medication. | Discovering pharmacotherapeutic combination | Hits of 0.39 for TuSDC-SC1 vs. 0.38 for TuSDCS, P < 0.001; MRR of 0.52 for TuSDC-SC1 vs. 0.49 for TuSDC-S, P < 0.001. Hits@1 of 0.4 for TuSDC-SM vs. 0.38 for TuSDC-S, P < 0.001; MRR of 0.52 for TuSDC-SM vs. 0.49 for TuSDC-S, P < 0.001 | Precision of 0.77 |
| **Machine Learning-Based prediction of Post-Treatment ambulatory blood pressure in patients with hypertension.** | 2023 | Hypertension | Retrospective | 1129 | 10.1080/08037051.2023.2209674 | Internal | CatBoost | Tree-based / Ensemble Methods | Clinical, laboratory, blood preasure data at baseline and treatment | Post- treatment Ambulatory blood preasure | Predicted vs. measured mean 24-hour systolic BP at follow-up was 8.4 ± 7.0 mm Hg (% difference of 6.6% ± 5.7%). CatBoost-predicted vs. the ABPM-measured changes in the mean 24-hour Systolic (r = 0.74) and diastolic (r = 0.68) | N.A. |
| **Causal Deep Neural Network-Based Model for First-Line Hypertension Management.** | 2023 | Hypertension | Retrospective | 16917 | 10.1016/j.mcpdig.2023.10.001 | Internal | deep neural network | Neural Networks | Clinical variables, vital signs, demographic, laboratory and medication. | Likelihood of a successful antihypertensive treatment 1 year from the start of treatment | Precision of 51.7%, recall of 44.4%, and F1 score of 47.8% | N.A. |
| **Machine-learning-based high-benefit approach versus conventional high-risk approach in blood pressure management.** | 2023 | Hypertension | Retrospective | 25247 | 10.1093/ije/dyad037 | External | Causal forest | Tree-based / Ensemble Methods | Clinical variables, demographic, laboratory and medication. | Identifying individuals with the highest estimated benefit from intensive treatment | Sample average treatment effect (95% CI High-benefit approach +9.36% (+8.33 to +10.44) vs High-risk approach +1.65% (+0.36 to +2.84) difference between these two approaches, +7.71% (6.79-8.67), P-value <0.001] | High-benefit approach +9.68% (+8.34 to +10.90) vs High-risk approach #1 +2.52% (+1.15 to +3.97) |
| **Evaluation of machine learning algorithms for renin-angiotensin-aldosterone system inhibitors associated renal adverse event prediction.** | 2023 | Hypertension | Retrospective | 409 | 10.1016/j.ejim.2023.05.021 | Internal | Random forest (RF), k-nearest neighbor (kNN), naïve Bayes (NB), extreme gradient boosting (xGB), support vector machine (SVM), neural network (NN), and logistic regression (LR) | Mixed | Clinical variables, demographic, laboratory and medication. | Renal adverse effects | kNN, RF, xGB and NN algorithms have the highest and similar AUC (≥ 98%), recall (≥ 94%), specifity (≥ 97%), precision (≥ 92%), accuracy (≥ 96%) and F1 statistics (≥ 94%) | N.A. |
| **Machine learning algorithms identify hypokalaemia risk in people with hypertension in the United States National Health and Nutrition Examination Survey 1999–2018.** | 2023 | Hypertension | Retrospective | 25326 | 10.1080/07853890.2023.2209336 | Internal | Logistic Regression, k-Nearest Neighbor, Random Forest, Recursive Partitioning and Regression Trees, and eXtreme Gradient Boosting | Tree-based / Ensemble Methods | Clinical variables, demographic, laboratory and medication. | Hypokalaemia risk | Random Forest:  AUROC (hypertension dataset:  0.73 [95%CI, 0.71-0.76]; CVD subgroup:  0.72 [95%CI, 0.66-0.78]) | N.A. |
| **A machine learning approach identifies modulators of heart failure hospitalization prevention among patients with type 2 diabetes: A revisit to the ACCORD trial.** | 2022 | Hypertension. Diabetes | Retrospective | 14984 | 10.1016/j.jdiacomp.2022.108287 | Internal | Causal forest, causal tree analysis. | Tree-based / Ensemble Methods | Clinical, demographic and laboratory test. | Heart Failure Hospitalizations | increased Heart Failure (HF) risk associated with intensive glycemic control (absolute risk change (ARC):  2.28 %, 95 % confidence interval (Confidence Interval (CI)):  0.69 % to 3.90 %; relative risk (RR): 1.57, 95 % Confidence Interval (CI):  1.15 to 2.20; P < 0.05) . baseline ALT at the lowest tertile (8-19 mg/dl) benefited from the intensive BP control for Heart Failure (HF) prevention (ARC:  -1.95 %, 95 % Confidence Interval (CI):  -4.06 % to 0.11 %; RR: 0.62. 95 % Confidence Interval (CI):  0.27 to 0.94; P < 0.05). | N.A. |
| **A preliminary evaluation of neural network analysis for pharmacodynamic modeling of the dosing of the hydroxymethylglutaryl coenzyme A-reductase inhibitors simvastatin and atorvastatin.** | 2002 | PK/PD | Retrospective | 17 | 10.1016/s0149-2918(02)85140-2 | Internal | NeuralSIM neural network | Neural Networks | Clinical, Laboratory and medication | Simvastatine or atorvastatine dosing | Data set 1:  Simvastatine R 0.89, 20% Accuracy 0.93%. Atorvastatine R 0.68, 20% Accuracy 0.62%.  Data set 2:  Simvastatine R 0.83, 20% Accuracy 0.97%. Atorvastatine R 0.92, 20% Accuracy 0.76% | N.A. |
| **Application of a Backpropagation Artificial Neural Network in Predicting Plasma Concentration and Pharmacokinetic Parameters of Oral Single-Dose Rosuvastatin in Healthy Subjects.** | 2020 | PK/PD | Retrospective | 209 | 10.1002/cpdd.809 | Internal | ANN | Neural Networks | Clinical, demographic, fasting/fed and laboratory parameters | Plasma concentration and pharmacokinetic parameters of rosuvastatin | Mean Squared Error (MSE):  0.00623. Magnitude of the gradient 0.0008071. Number of validation checks  0. Correlation coefficient  0.954 . There are no significant differences (P > .05) between the measured and predicted data. | N.A. |
| **Applying an artificial neural network to warfarin maintenance dose prediction.** | 2004 | Thrombosis | Retrospective | 148 | 15609884 | Internal | ANN | Neural Networks | Clinical, demographic and medication. | Maintenance dosis of Warfarin | Correct maintenance dose:  75% in the Artificial Neural Network (ANN) group vs. 57% in the control group in the linear model (p = 0.275, T-Student). | N.A. |
| **Use of artificial neural network to predict warfarin individualized dosage regime in Chinese patients receiving low-intensity anticoagulation after heart valve replacement** | 2014 | Thrombosis | Retrospective | 1093 | 10.1016/j.ijcard.2014.08.062 | Internal | Artificial neural network (ANN), multiple linear regression (MLR) | Neural Networks | Clinical variables, demographic, laboratory and medication. | Maintenance dosis of Warfarin | ForArtificial Neural Network (ANN),theideal predicted percentages accoun tfor 65.93–68.50% and the MAE isl ess than 0.115mg/day, while for MLR, the ideal predicted percentages is 45.42–66.30% and the MAE is less than 0.595mg/day | N.A. |
| **Prediction of optimal warfarin maintenance dose using advanced artificial neural networks** | 2014 | Thrombosis | Retrospective | 377 | 10.2217/pgs.13.212 | Internal | artificial neural networks (ANNs) | Neural Networks | Clinical variables, demographic and genetics. | Maintenance dosis of Warfarin | Average absolute error of 5.7 mg. In the subset of patients requiring ≤21 mg and 21-49 mg  the absolute error was 3.86 mg and 5.45 with a high percentage of subjects being correctly identified (71 and 73%, respectively) | N.A. |
| **Ensemble of machine learning algorithms using the stacked generalization approach to estimate the warfarin dose** | 2018 | Thrombosis | Retrospective | 5743 | 10.1371/journal.pone.0205872 | Internal | Gradient boosting trees (GBT), neural networks (NN), ridge regression (RR), random forest (RF), and extremely randomized trees (ET), support vector regression (SV), multivariate linear regression (MLR), K nearest neighbors (KN) | Mixed | Clinical variables, demographic, laboratory, medication and genetics. | Maintenance dosis of Warfarin | Stack 1 MAE (9.31 (7.86-8.76) 95% Confidence Interval (CI) p<0.001). Withing 20% 47.85 (45.43-50.28) 95% Confidence Interval (CI) p<0.001). Stack 2 MAE (8.31 (7.87-8.76) 95% Confidence Interval (CI) p<0.001). Withing 20% 47.81 (45.44-50.19) 95% Confidence Interval (CI) p<0.001) | N.A. |
| **Warfarin maintenance dose Prediction for Patients undergoing heart valve replacement- a hybrid model with genetic algorithm and Back-Propagation neural network** | 2018 | Thrombosis | Retrospective | 14231 | 10.1038/s41598-018-27772-9 | External | Back-Propagation neural network (BP-GA) | Neural Networks | Clinical variables, demographic, laboratory and echocardiogram. | Maintenance dosis of Warfarin | MAE:   0.383 mg/d  (p < 0.05). RMean Squared Error (MSE)  0.664 mg/d | MAE:   0.370 mg/d  (p < 0.05). RMean Squared Error (MSE) (0.656 mg/d) |
| **Stepped-wedge randomised trial to evaluate population health intervention designed to increase appropriate anticoagulation in patients with atrial fibrillation.** | 2019 | Thrombosis | Prospective | 1727 | 10.1136/bmjqs-2019-009367 | External | – | Other | Clinical, laboratory and medication. | Eligible patients prescribed an OAC with previous AF diagnosis | Intervention:  4.1% vs usual care:  4.0%, p=0.86. | N.A. |
| **New artificial intelligence prediction model using serial prothrombin time international normalized ratio measurements in atrial fibrillation patients on vitamin K antagonists: GARFIELD-AF** | 2020 | Thrombosis | Retrospective | 4708 | 10.1093/ehjcvp/pvz076 | Internal | ANN | Neural Networks | Laboratory test | Major bleed, stroke/systemic embolism and all-cause death | The model's c-statistic for predicting major bleed, stroke/SE, and all-cause death was 0.75, 0.70, and 0.61, respectively. | N.A. |
| **Machine Learning Algorithm for Predicting Warfarin Dose in Caribbean Hispanics Using Pharmacogenetic Data** | 2020 | Thrombosis | Retrospective | 190 | 10.3389/fphar.2019.01550 | Internal | Random forest regression (RFR), vector regression (SVR), multivariate adaptive splines (MARS), artificial neural networks (ANN), recursive partitioning (RPART),  K-nearest neighbor for K from 1 to 3, reduces error tree classifier (REPT). | Mixed | Clinical variables, demographic and genetics. | Maintenance dosis of Warfarin | Normal group dose requirement:  RFR  (MAE = 2.91 mg/week). Sensitive group:  SVR  (MAE of 4.79 mg/week). Resistant group:  MARS (MAE = 7.22 mg/week). | N.A. |
| **An Adapted Neural-Fuzzy Inference System Model Using Preprocessed Balance Data to Improve the Predictive Accuracy of Warfarin Maintenance Dosing in Patients After Heart Valve Replacement** | 2021 | Thrombosis | Retrospective | 15108 | 10.1007/s10557-021-07191-1 | External | Neural-fuzzy inference system (ANFIS) | Neural Networks | Clinical variables and laboratory parameters. | Maintenance dosis of Warfarin | Accuracy:   balanced ANFIS model  75.31%, and  imbalanced ANFIS model 78.16%. MAE  was 0.421 and 0.368 respectively. | Accuracy:  balanced ANFIS 73.46% and imbalanced ANFIS 74.39%. MAE:  balanced model 0.422, and  imbalanced model 0.370 |
| **Predicting Range of Initial Warfarin Dose Based on Pharmacometabolomic and Genetic Inputs** | 2021 | Thrombosis | Retrospective | 160 | 10.1002/cpt.2407 | Internal | Partial least-squares model. Two-dimensional linear discriminant analysis-multinomial logit model. IniWarD. | Linear / Regularized Models | Clinical variables, demographic, medication, laboratory and genetics. | Variation in warfarin response and suggest an initial daily dose range | Partial least-square model Coefficient of determination (R²) = 0.753 for the training set, 0.643 for the test set. Two-dimensional linear discriminant analysis-multinomial logit model  accuracy of 91% for the training set and 90% for the test set | N.A. |
| **Development of a system to support warfarin dose decisions using deep neural networks** | 2021 | Thrombosis | Retrospective | 34987 | 10.1038/s41598-021-94305-2 | Internal | Recurrent Neural Network | Neural Networks | Clinical variables, demographic and laboratory test. | PT-INR prediction and warfarin maintenance dose | ML algorithm:  10,650/12,673 cases (84.0%), expert physicians:  1647/2000 cases (81.9%), P = 0.014. Warfarin dose-PT INR tables were within 0.3 of actual value in 450/842 cases (53.4%) | N.A. |
| **A Clinical Prediction Model to Predict Heparin Treatment Outcomes and Provide Dosage Recommendations: Development and Validation Study** | 2021 | Thrombosis | Retrospective | 5156 | 10.2196/27118 | Internal | ANN | Neural Networks | Clinical variables, demographic, laboratory and medication. | Heparin dosage recommendations | F1 scores 0.887 (MIMIC III) and 0.925 (PUMCH).  For 72.2 % (1240/1718) of the subtherapeutic samples in MIMIC III and 64.7% (281/434) of the subtherapeutic samples in PUMCH, the model recommended a higher heparin dosage than the clinicians did. For 80.9% (504/623) of the supratherapeutic samples in MIMIC III and 76.7% (277/361) of the supratherapeutic samples in PUMCH, the model recommended a lower heparin dosage than the clinicians did. | N.A. |
| **Comparative Effectiveness of Machine Learning Approaches for Predicting Gastrointestinal Bleeds in Patients Receiving Antithrombotic Treatment** | 2021 | Thrombosis | Retrospective | 306463 | 10.1001/jamanetworkopen.2021.10703 | Internal | Cox proportional hazards regression (RegCox), XGBoost, random survival forests (RSF) | Tree-based / Ensemble Methods | Clinical variables, demographic, laboratory and medication. | Gastrointestinal bleeding | AUC:  RegCox 0.67 at 6 months and 0.66 at 12 months. XGBoost 0.67 at 6 months and 0.66 at 12 months. RSF 0.62 at 6 months and 0.60 at 12 months | N.A. |
| **An ensemble learning based framework to estimate warfarin maintenance dose with cross-over variables exploration on incomplete data set** | 2021 | Thrombosis | Retrospective | 377 | 10.1016/j.compbiomed.2021.104242 | Internal | LightGBM, XGBoost, CatBoost, GBDT, RandomForest, SVM, KNN, Lasso Regression, Ridge Regression, MLR and a state-of-art deep learning method | Mixed | Clinical variables, demographic, laboratory, genetics and medication. | Maintenance dosis of Warfarin | LightGBM outperforms others by a large margin, with the average Coefficient of determination (R²) of 0.750, Mean Squared Error (MSE) of 0.470, RMean Squared Error (MSE) of 0.681, MAE of 0.436 over the 16 single variables | N.A. |
| **Evaluating warfarin dosing models on multiple datasets with a novel software framework and evolutionary optimisation** | 2021 | Thrombosis | Retrospective | 4621 | 10.1016/j.jbi.2020.103634 | Internal | Linear Regression, Support Vector Regression, among others. | Kernel-based Methods | Clinical variables, demographic and medication. | Maintenance dosis of Warfarin | LR (MAE 10.98, PW20 34.08) and SV (MAE 10.93, PW2034.40) | N.A. |
| **Combining mathematical modeling and deep learning to make rapid and explainable predictions of the patient-specific response to anticoagulant therapy under venous flow.** | 2022 | Thrombosis | Retrospective | 7000 | 10.1016/j.mbs.2022.108830 | Internal | ANN | Neural Networks | Laboratory test | Response to anticoagulant therapy under flow, thrombin generation/formation | Accuracy 96% | N.A. |
| **Evaluation of supervised machine learning algorithms in predicting the poor anticoagulation control and stable weekly doses of warfarin** | 2022 | Thrombosis | Retrospective | 1962 | 10.1007/s11096-022-01471-y | Internal | Decision tree classification; Regression tree analysis (CART), Chi-square automatic interaction detector | Tree-based / Ensemble Methods | Clinical, demographic, medication, laboratory and genetics. | Maintenance dosis of Warfarin | Area under the receiver-operating characteristics curve (AUROC) = 0.53. Percentage of patients within 20% of the actual dose = 38.2%, root mean squared error (RMean Squared Error (MSE)) = 13.6. CHAID algorithm, the percentage of patients within 20% of the actual dose was 49%, RMean Squared Error (MSE) 13,4. | N.A. |
| **Development and validation of a mobile application based on a machine learning model to aid in predicting dosage of vitamin K antagonists among Indian patients post mechanical heart valve replacement** | 2022 | Thrombosis | Retrospective | 1560 | 10.1016/j.ihj.2022.10.002 | Internal | Linear Regression, Support Vector Regression, Logistic Regression, Multi Layer Perceptron | Kernel-based Methods | Clinical variables, demographic, medication, and laboratory parameters. | Maintenance dosis of Warfarin | Support Vector Regression:  mean square error of 0.41 and Coefficient of determination (R²) of 0.955 | N.A. |
| **Warfarin anticoagulation management during the COVID-19 pandemic: The role of internet clinic and machine learning** | 2022 | Thrombosis | Retrospective | 241 | 10.3389/fphar.2022.933156 | Internal | K-nearest neighbors (KNN), support vector machine (SVM), random forest classifier (RFC), eXtreme Gradient Boosting (XGBoost), and Light Gradient Boosting Machine (LightGBM) | Mixed | Clinical variables, demographic, medication and laboratory. | Anticoagulation quality and incidence of adverse events | XGBoost:  sensitivity (76.7%),AUC(0.808), and accuracy(0.767). KNN:  sensitivity (63.16%),AUC(0.617), and accuracy(0.548).  SVM:  sensitivity (79%),AUC(0.801), and accuracy(0.644).  RFC:  sensitivity (73.7%),AUC(0.786), and accuracy(0.767).  LightGBM:  sensitivity (73.7%),AUC(0.795), and accuracy(0.699). | N.A. |
| **Predicting Therapeutic Response to Unfractionated Heparin Therapy: Machine Learning Approach** | 2022 | Thrombosis | Retrospective | 3019 | 10.2196/34533 | External | XGBoost, LightGBM, generalized linear models, TensorFlow, RuleFit and FTRL | Tree-based / Ensemble Methods | Clinical variables, demographic, medication and laboratory. | aPTT within 12 hours after a specified bolus and maintenance dose of unfractionated heparin | LightGBM models with an RMean Squared Error (MSE):  accuracy of 0.599 and an AUC of 0.735. | Accuracy of 0.568 and AUC of 0.724 |
| **Optimizing the dynamic treatment regime of in-hospital warfarin anticoagulation in patients after surgical valve replacement using reinforcement learning** | 2022 | Thrombosis | Retrospective | 10408 | 10.1093/jamia/ocac088 | Internal | ANN | Neural Networks | Clinical variables, demographic, medication, echocardiogram and laboratory parameters. | Maintenance dosis of Warfarin | Responder ratio 41.6% versus 80.8% (relative risk [RR], 0.51; 95% confidence interval [Confidence Interval (CI)], 0.48-0.55). Safety responder ratio:  83.1% versus 99.5% (RR, 0.83; 95% Confidence Interval (CI), 0.81-0.86). Target responder ratio:  49.7% versus 81.1% (RR, 0.61; 95% Confidence Interval (CI), 0.58-0.65) | N.A. |
| **Can Machine Learning from Real-World Data Support Drug Treatment Decisions? A Prediction Modeling Case for Direct Oral Anticoagulants** | 2022 | Thrombosis | Retrospective | 29901 | 10.1177/0272989X211064604 | Internal | Random forest | Tree-based / Ensemble Methods | Clinical variables, demographic and medication. | Strokes and major bleeding | Reduced strokes:  C-for-benefit:  0.56; 95% confidence interval [0.52; 0.60]. Absolute risk reduction ARR with Apixaban:  1.69 % [0.39; 2.97]. ARR with Rivaroxaban (ARR: 20.88% [22.93; 1.21]. Absolute risk difference ARD of the composite clinical outcome:  -0.78 % [-1.40; -0.03] | N.A. |
| **Determining the adjusted initial treatment dose of warfarin anticoagulant medicine using kernel-based support vector regression** | 2022 | Thrombosis | Retrospective | 5700 | 10.1016/j.cmpb.2021.106589 | Internal | Kernel-based version of the least square support vector regression | Kernel-based Methods | Clinical variables, demographic, medication, laboratory and genetics. | Initial treatment Warfarin dose | MAE 8,0068 (IC 95% 0.086258 - 0.091698). Coefficient of determination (R²) 0.68. | N.A. |
| **Effectiveness of an artificial intelligence clinical assistant decision support system to improve the incidence of hospital-associated venous thromboembolism: a prospective, randomised controlled study.** | 2023 | Thrombosis | Prospective | 19785 | 10.1136/bmjoq-2023-002267 | External | – | Other | Clinical, laboratory, imaging and medication | Venous thromboembolism | Relative Reduction of 46.00% (OR:  0.55, 95%Confidence Interval (CI) (0.34 to 0.88) in venous thromboembolic events. Similar frequency was observed in deep veint thrombosis events (OR:  0.50, 95%Confidence Interval (CI) (0.30 to 0.83) | N.A. |
| **Machine learning models to predict the warfarin discharge dosage using clinical information of inpatients from South Korea** | 2023 | Thrombosis | Retrospective | 4059 | 10.1038/s41598-023-49831-6 | External | XGBoost; artificial neural network; random forest; linear regression. | Mixed | Clinical variables, vital sign, demographic and medication. | Maintenance dosis of Warfarin | Mean absolute error (MAE):  XGBoost, 0.9; artificial neural network, 0.9; random forest, 1.0; linear regression, 1.0; and physicians, 1.3 | MAEs:  XGBoost, 1.9; Artificial Neural Network (ANN), 2.0; random forest, 1.8; linear regression, 1.8;  physicians, 1.8. |
| **XGBoost-based machine learning test improves the accuracy of hemorrhage prediction among geriatric patients with long-term administration of rivaroxaban** | 2023 | Thrombosis | Retrospective | 892 | 10.1186/s12877-023-04049-z | External | Random forest. XGBoost | Tree-based / Ensemble Methods | Clinical, demographic, medication and laboratory parameters. | Bleeding | XGBoost AUC of 0.776 (95% Confidence Interval (CI):  0.687, 0.864) with an accuracy 0.771, sensitivity 0.804, precision 0.921, recall 0.904, and F-1 of 0.859.  Random forest  AUC of 0.672 | XGBoost AUC of 0.689 (95% Confidence Interval (CI):  0.518, 0.860); |
| **Identifying treatment heterogeneity in atrial fibrillation using a novel causal machine learning method** | 2023 | Thrombosis | Retrospective | 34569 | 10.1016/j.ahj.2023.02.015 | Internal | Causal ML method. Causal tree | Tree-based / Ensemble Methods | Clinical variables, demographic and laboratory parameters. | Ischemic stroke, intracranial hemorrhage, and all-cause mortality | The causal ML method identified 5 subgroups with variables favoring apixaban over dabigatran; 2 subgroups favoring apixaban over rivaroxaban; 1 subgroup favoring dabigatran over rivaroxaban; and 1 subgroup favoring rivaroxaban over dabigatran in terms of risk reduction of the primary endpoint. No subgroup favored warfarin and most dabigatran vs warfarin users favored neither drug. | N.A. |
| **Automated warfarin dose prediction for Asian, American, and Caucasian populations using a deep neural network** | 2023 | Thrombosis | Retrospective | 6000 | 10.1016/j.compbiomed.2023.106548 | Internal | ANN | Neural Networks | Clinical variables, demographic, medication, laboratory and genetics. | Maintenance dosis of Warfarin | MAE of 7.6 mg/week and a relatively low mean percentage of error of 40.9% in Asians, 14.2 mg/week MAE and 36.9% in African Americans, and 12.7 mg/week MAE and 45.4% mean percentage of error in White Caucasians | N.A. |
| **Integrated Machine Learning Decision Tree Model for Risk Evaluation in Patients with Non-Valvular Atrial Fibrillation When Taking Different Doses of Dabigatran** | 2023 | Thrombosis | Retrospective | 11802 | 10.3390/ijerph20032359 | Internal | Naive Bayes, random forest (RF), classification and regression tree (CART), and extreme gradient boosting (XGBoost) | Tree-based / Ensemble Methods | Clinical variables, demographic and laboratory parameters. | Suggestive rules to clinicians managing | AUC:  RF (0.764 for 110 mg; 0.747 for 150 mg); XGBoost (0.708 for 110 mg; 0.761 for 150 mg); Logistic regression (benchmark model; 0.683 for 110 mg; 0.739 for 150 mg) | N.A. |
| **Computational Structural Validation of CYP2C9 Mutations and Evaluation of Machine Learning Algorithms in Predicting the Therapeutic Outcomes of Warfarin** | 2023 | Thrombosis | Retrospective | – | 10.2174/1389200224666230705124329 | Internal | – | Other | Clinical variables, demographic, laboratory and genetics. | Predicting the poor anticoagulation status and stable warfarin dose | ML algorithms revealed CYP2C9 to be the most important predictor | N.A. |
| **Improved stacking ensemble learning based on feature selection to accurately predict warfarin dose** | 2024 | Thrombosis | Retrospective | 641 | 10.3389/fcvm.2023.1320938 | Internal | Heuristic-stacking ensemble learning | Tree-based / Ensemble Methods | Clinical, Demographic, medication and genetics. | Maintenance dosis of Warfarin | Accuracy  73.44%; degree of regression fit Coefficient of determination (R²) reaching 0.87. | N.A. |

ACS: Acute Coronary Syndrome, ANN: Artificial Neural Network, AUC: Area Under the Curve, AUROC: Area Under the Receiver Operating Characteristic Curve, AUPRC: Area Under the Precision-Recall Curve, BPs: Blood Pressures, CART: Classification and Regression Tree, CI: Confidence Interval, DPP-4is: Dipeptidyl Peptidase-4 Inhibitors, GLP-1RA: Glucagon-Like Peptide-1 Receptor Agonist, GP: Gaussian Process, HF: Heart Failure, HR: Hazard Ratio, INR: International Normalized Ratio, IQR: Interquartile Range, KNN: K-Nearest Neighbors, LASSO: Least Absolute Shrinkage and Selection Operator, LDA: Linear Discriminant Analysis, LQTS: Long QT Syndrome, LVEF: Left Ventricular Ejection Fraction, MAE: Mean Absolute Error, MACE: Major Adverse Cardiovascular Events, MI: Myocardial Infarction, MSE: Mean Squared Error, NB: Naive Bayes, OR: Odds Ratio, PK/PD: Pharmacokinetics/Pharmacodynamics, QDA: Quadratic Discriminant Analysis, R²: Coefficient of determination, RF: Random Forest, ROC: Receiver Operating Characteristic, SGLT2: Sodium–Glucose Co-Transporter-2 Inhibitors, SVM: Support Vector Machine, XGBoost: Extreme Gradient Boosting.
